# Active evolution of memory B-cells specific to viral gH/gL/pUL128/130/131 pentameric complex in healthy subjects with silent human cytomegalovirus infection

**DOI:** 10.18632/oncotarget.18359

**Published:** 2017-06-03

**Authors:** Lin Xia, Aimin Tang, Weixu Meng, Daniel C. Freed, Linling He, Dai Wang, Fengsheng Li, Leike Li, Wei Xiong, Xun Gui, Robbie D. Schultz, Haotai Chen, Xi He, Ryan Swoyer, Sha Ha, Yaping Liu, Charles D. Morris, Yu Zhou, I-Ming Wang, Qinjian Zhao, Wenxin Luo, Ningshao Xia, Amy S. Espeseth, Daria J. Hazuda, Richard E. Rupp, Alan D. Barrett, Ningyan Zhang, Jiang Zhu, Tong-Ming Fu, Zhiqiang An

**Affiliations:** ^1^ Texas Therapeutics Institute, The Brown Foundation Institute of Molecular Medicine, University of Texas Health Science Center at Houston, Houston, TX, USA; ^2^ Merck Research Laboratories, Merck & Co., Inc., Kenilworth, NJ, USA; ^3^ State Key Laboratory of Molecular Vaccinology and Molecular Diagnostics, National Institute of Diagnostics and Vaccine Development in Infectious Diseases, Xiamen University, Xiamen, China; ^4^ Department of Immunology and Microbial Science, The Scripps Research Institute, La Jolla, CA, USA; ^5^ Sealy Center for Vaccine Development, University of Texas Medical Branch, Galveston, TX, USA; ^6^ Center for HIV/AIDS Vaccine Immunology and Immunogen Discovery, The Scripps Research Institute, La Jolla, CA, USA; ^7^ Department of Integrative Structural and Computational Biology, The Scripps Research Institute, La Jolla, CA, USA

**Keywords:** human cytomegalovirus, human antibodies, B-cell repertoire, antiviral antibody, neutralization

## Abstract

Human cytomegalovirus (HCMV) can cause life-threatening infection in immunosuppressed patients, and *in utero* infection that may lead to birth defects. No vaccine is currently available. HCMV infection in healthy subjects is generally asymptomatic, and virus persists as latent infection for life. Host immunity is effective against reactivation and super-infection with another strain. Thus, vaccine candidates able to elicit immune responses similar to those of natural infection may confer protection. Since neutralization is essential for prophylactic vaccines, it is important to understand how antiviral antibodies are developed in natural infection. We hypothesized that the developmental path of antibodies in seropositive subjects could be unveiled by interrogating host B-cell repertoires using unique genetic signature sequences of mAbs. Towards this goal, we isolated 56 mAbs from three healthy donors with different neutralizing titers. Antibodies specific to the gH/gL/pUL128/130/131 pentameric complex were more potent in neutralization than those to gB. Using these mAbs as probes, patterns of extended lineage development for B-cells and evidence of active antibody maturation were revealed in two donors with higher neutralizing titers. Importantly, such patterns were limited to mAbs specific to the pentamer, but none to gB. Thus, memory B-cells with antiviral function such as neutralization were active during latent infection in the two donors, and this activity was responsible for their higher neutralizing titers. Our results indicated that memory B-cells of neutralizing capacity could be frequently mobilized in host, probably responding to silent viral episodes, further suggesting that neutralizing antibodies could play a role in control of recurrent infection.

## INTRODUCTION

Human cytomegalovirus (HCMV) is a prototype beta-herpesvirus, prevalent in over 50% adult population worldwide [1]. In healthy subjects, HCMV infection does not cause discernible clinical symptoms [2], and the virus establishes latent infection that persists throughout the life of the host [3]. Although the host immune system is incapable of eliminating the latent virus, can effectively control subsequent viral infections, also called recurrent infections, which include viral reactivation from latency and super-infection with another strain [4-6]. When the host immune system is compromised such as in those transplant recipients under immunosuppression, HCMV infection can be serious and even lead to life-threatening disease [7, 8]. Fetuses with an immature immune system are also vulnerable to HCMV; *in utero* HCMV infection has been identified as a leading cause of birth defects in the United States, with variety of neuronal developmental sequelae including sensorineural hearing loss, microcephaly, and mental retardation [9, 10]. There are no options currently available for prevention of congenital HCMV, despite the fact that development of a prophylactic vaccine has been assigned to the highest category of vaccine priority by the Institute of Medicine since 1999 [11, 12].

Natural immunity is effective against subsequent HCMV infection. HCMV seropositive transplant recipients are more resistant to recurrent infection and HCMV disease as compared to HCMV seronegative recipients with transplants from HCMV seropositive organ donors [13]. Similarly, HCMV seropositive women are protected against super-infection of the virus from their children in daycare as compared with HCMV seronegative women [14]. Importantly, maternal HCMV seropositive status prior to pregnancy is associated with ∼69% reduction of maternal-fetal transmission [15, 16]. These observations support the notion that a prophylactic vaccine is feasible if it can elicit immune responses similar to those of natural immunity. Live attenuated AD169 and Towne vaccines, and a recently described replication-defective virus vaccine [17], were all developed based on this concept. Thus, an in-depth understanding of the attributes of adaptive immunity in HCMV seropositive subjects will be imperative for development of successful vaccine candidates against this viral disease, both for rational vaccine design and assessment of vaccine-induced immune responses in clinical studies.

HCMV is a complex virus capable of expressing more than 160 viral proteins during its life cycle [18], all of which can be targeted by host immune responses. Sylwester and coworkers have shown that more than 70% of total antigens can be recognized by human T-cells from a cohort of 33 HCMV seropositive donors [19]. In addition, HCMV is known to expand host T-cell pools of effector-memory phenotypes [20], and as many as 10% of host CD4^+^ and CD8^+^ T-cells can be dedicated to HCMV in healthy subjects [19]. Such expansion is termed memory T-cell inflation, and it has been linked to the role of T-cells in controlling recurrent viral infection [20], as demonstrated in transplant recipients with frequent viral reactivation [21, 22]. On the other hand, neutralizing antibodies are important for prevention of HCMV acquisition, as either primary infection in seronegative individuals or super-infection in seropositive ones. Thus, neutralization capability is an essential quality of immune responses to a prophylactic vaccine. Understanding anti-HCMV B-cells and monoclonal antibodies (mAbs) in the context of natural infection would provide valuable insights for vaccine design, as exemplified by the recent design and evaluation of a subunit vaccine [23]. However, humoral immune responses to HCMV have yet to be comprehensively studied. Natural HCMV infection can induce robust antibody responses, with neutralizing titers well sustained across all age groups [24]. The viral pentameric complex, composed of glycoprotein H (gH), glycoprotein L (gL), pUL128, pUL130 and pUL131 (or pUL131a) is recognized as an important target for neutralization against viral infection of epithelial cells [25-29]. However, the antigen specificity of overall neutralizing antibodies in seropositive donors has not been investigated at clonal levels. In addition, it is yet to be determined how host neutralizing antibodies are shaped by natural HCMV infection, a question that could be addressed by analyzing lineage development of the memory B-cells that produce antiviral antibodies.

Towards the goal of systematic analysis of human B-cell responses to HCMV, we first established a panel of 56 mAbs from three healthy donors with high, medium and low serum neutralizing titers to HCMV. Since memory B-cells can acquire somatic mutations when exposed to recall antigens, we hypothesized that the unique genetic identities of HCMV antibodies, such as the complementarity determining regions (CDRs), could be used to probe the evolutionary paths of these memory B-cells in the donors’ B-cell repertoires. Here we demonstrated the genetic imprints of the 56 mAbs in their respective B-cell repertoires, and further identified donor-specific antibody lineage patterns indicative of active memory B-cell evolution. Interestingly, the extended lineage development was only evident in B-cells producing antibodies specific to the pentameric complex, a viral antigen targeted by potent neutralizing mAbs [25, 27, 30, 31]. By contrast, lineage development was not detected in B-cells producing antibodies to glycoprotein B (gB), an antigen less frequently targeted by potent neutralizing antibodies in human subjects [27, 32]. Therefore, active B-cell evolution could occur in human subjects with silent recurrent viral episodes, and the maturation was limited to antibodies targeting the key antigen for neutralization.

## RESULTS

### Isolation and functional characterization of HCMV-specific antibodies

We previously determined the geometric mean of viral neutralization titers, reported as reciprocal serum dilutions to block 50% input virus entry in human epithelial ARPE-19 cells (NT_50_), to be ∼7000 in a cohort of 360 healthy HCMV seropositive women. The eightieth and twentieth percentiles of the NT_50_ titers for this cohort are calculated as 17200 and 3300, respectively [24]. Accordingly, we identified three healthy adult donors with NT_50_ titers of 13500, 6000, and 1500, representing high, medium, and low in the spectrum of the NT_50_ titers (Table 1). All three donors reported no clinical history of HCMV infection or disease.

**Table 1:** Summary of donor selection, memory B-cell culture and antibody cloning

	donor 1	donor 2	donor 3
Donor selection			
Gender	Male	Male	Female
Approximate age^1^	40s	40s	30s
NT_50_^2^	13500	6000	1500
Memory B cell culture screening			
Total number of culture wells^3^	19200	19200	19200
Estimated IgG positive wells^4^	9965	11059	7360
Hits	191	118	78
Neutralizing positive wells^5^	21	11	4
Virion ELISA positive wells^6^	176	110	75
Binding and neutralizing wells^7^	6	3	1
Antibodies from memory cell cultures			
Heavy chain cloned	183	106	73
Light chain cloned	182	109	72
mAbs expressed and characterized	66	37	40
confirmed mAbs	23	17	16
neutralizing mAbs^8^	9	3	2

^1^donor’s age was approximated.

^2^NT_50_: reciprocal serum dilution to neutralize 50% input virus in ARPE-19 cells

^3^B-cells were cultured at approximately 1.4 cells per well in 200 96-well plates.

^4^IgG positive wells (with OD450nm reading > 0.1) were estimated based on random sampling of 8-10 96-well plates for each subject (Figure S1B).

^5^Neutralizing positive well (hit) was defined as greater than 80% reduction in fluorescence signal by HCMV IE antibody staining in neutralization assay.

^6^Virion ELISA positive well (hit) was defined as OD450nm reading > 0.1 using AD169rev as antigen.

^7^B-cell culture wells screened positive for both neutralizing and virion ELISA.

^8^Neutralizing mAb was defined as those with IC_50_ below 1 μg/mL

Methods for culturing single memory B-cells, cloning immunoglobulin (Ig) genes, and identifying functional mAbs are outlined in Supplementary Figure S1A. The efficiency of single memory B-cell culture was confirmed by sampling human IgG production in culture supernatants, which showed separation of IgG^+^ and IgG^-^ B-cell cultures (Supplementary Figure S1B). The IgG^+^ B-cell cultures contained ≥0.1 µg/mL IgG, sufficient for screening antibodies for binding and neutralizing HCMV [33]. A culture would be scored as a hit if it was shown to have a specific binding signal in ELISA or more than 80% reduction of HCMV viral entry (Table 1). Viral neutralization assays were conducted in human epithelial cells (ARPE-19), which were about 8-10-fold more sensitive in detecting antiviral activity than assays using fibroblast cells [34, 35]. Importantly, host antiviral activity at mucosal epithelium is relevant to protection against HCMV acquisition. In addition, our previous experience in cloning antibodies from hybridoma cultures from a vaccinate rabbit showed that out of total 45 antibodies, all 25 neutralizing mAbs have antiviral activity in epithelial cells, but only 16 have shown neutralizing activity in fibroblast cells (31). Thus, using fibroblast cells for screening may miss some neutralizing hits.

The B-cell cultures from donor 1 yielded 191 hits, approximately 2% of the total IgG^+^ B-cell cultures (Table 1). Among the 191 hits, 21 cultures showed neutralizing activities, 176 were positive in HCMV ELISA, but only six tested positive in both assays. The cultures from donor 2 and donor 3 yielded 118 and 78 hits respectively, accounting for approximately 1% of the total IgG^+^ memory B-cell cultures (Table 1). The number of HCMV-specific hits for each donor correlated with their serum neutralizing titers, with the linear regression analysis revealing a correlation coefficient (*r*) of 0.99 and a *p* value of 0.015.

Variable domains of Ig genes (V_H_ and V_L_) were cloned from the cultures of hits (Supplementary Figure S2) with over 93% efficiency (Table 1). Next, we paired V_H_ and V_L_ genes from the same B-cell cultures. Then, to confirm the antibody function from the initial screening, we expressed 352 V_H_/V_L_ combinations as human IgG_1_ antibodies. Antibodies of V_H_/V_L_ pairs with confirmed activity were purified and assayed for quantitative neutralizing activity (IC_50_), defined as the concentration necessary to achieve 50% viral entry inhibition, and relative binding affinity to virus (virus-specific EC_50_), defined as the IgG concentration required to achieve 50% of maximal signal in virion ELISA (Supplementary Figure S3A, B, C). A total of 56 antibodies with unique amino acid sequences were identified. Each antibody was named with identification codes of the donors and the particular heavy/light chain sequence combinations (Table 2). Representative sets of these mAbs were plotted for their virus-specific EC_50_ values (*x*-axis) against IC_50_ values (*y*-axis) (Supplementary Figure S3D, S3E, S3F).

**Table 2:** Functional and genetic properties of HCMV-specific antibodies

mAb ID	Functions assessed with whole virion		mAb gene family usage		CDR3 amino acids		Relative bind affinity to recombinant HCMV antigens	
	Virus-specific EC50 (μg/ml)	Neutralization IC50 (μg/ml)	Heavy chain	Light chain	CDR3H	CDR3L	gB EC50 (μg/ml)	Pentamer EC50 (μg/ml)
1-15		0.0009	IGHV3-11*04	IGLV3-21*01	ARDSYSKLVDIEAIEAFDI	QVWDRTSDHVV		0.02
1-32		0.02	IGHV3-30*04	IGKV1-33*01	ARDMRYYYDSNGHYRNRYGMDV	QQYENLFT		0.002
1-36	6.6		IGHV3-30*04	IGLV6-57*01	ARDMRYYYDSNGHYRNRYGMDV	QSYDSTSQV		5.7
1-64		0.001	IGHV3-11*04	IGLV3-21*01	ARDSYSKLVDIEAIEAFDI	QVWDRTSDHVV		0.02
1-85		0.0009	IGHV3-11*04	IGLV3-21*01	ARDSYSKLVDIEAIEAFDI	QVWDRHGDHVV		0.01
1-103	0.2	0.004	IGHV4-39*01	IGKV1-9*01	ARRIRGYSGTYD	QQLNN		0.002
1-125	1.4	0.0009	IGHV3-11*04	IGLV3-21*01	ARDSYSKLVDIVAIEAFDL	QVWDSSSARLV		0.007
1-143	0.2	0.004	IGHV4-39*01	IGKV1-9*01	ARRIRGYSGTYD	QQLNN		0.002
1-150		0.001	IGHV3-11*04	IGLV3-21*01	ARDSYSKLADIEATEAFDV	QVWDSGSDRVV		0.006
1-155	0.1		IGHV1-69*01	IGLV2-11*01	AGVRVAGGDNWFDP	CSYAGNYTFYI	0.0009	
1-175		0.001	IGHV3-11*04	IGLV3-21*01	ARDSYSKLVEIEAIEAFDV	QVWDRQTDHVV		0.01
1-179	7		IGHV1-3*01	IGLV3-19*01	ARSIYYYGSGSYEEPDAFDI	NSRDSSANHWV		
1-189	0.2		IGHV1-69*01	IGKV4-1*01	AREKGGWYCDL	QQYYSFPRT	0.003	
1-190	0.07		IGHV1-69*01	IGKV4-1*01	ARDPHYGFFLH	QQYYSPPYT	0.002	
1-191	0.1		IGHV1-69*01	IGKV4-1*01	ARATPGYYFDY	QQYYSPPWT	0.002	
1-192	0.06		IGHV1-69*06	IGKV4-1*01	ARRRDNYYFDF	QQYYSTLLT	0.002	
1-193	0.01	1	IGHV1-18*01	IGKV2-30*01	ARDHVPTFLWVGQSLHSPDFDY	MQGTHWPWT	0.003	
1-194	0.006	0.7	IGHV1-18*01	IGKV2-30*01	AKDHMPTMFLLGGSVHSPDFDY	MQGTHWPWT	0.001	
1-223	0.4	8.3	IGHV1-69*03	IGLV2-23*01	YCATDFQGTYDYVWGGWGLFDN	CSYAGRRTVV	0.04	
1-224	0.02	6.4	IGHV1-69*03	IGKV3-20*01	YCATDFQGTYDYVWGGWGLFDN	QQYGTSLT	0.001	
1-228	0.06		IGHV3-13*01	IGKV1-33*01	ARGRDTPFDV	QQYEDVPLT	0.0009	
1-235	0.004		IGHV4-31*03	IGKV4-1*01	ACQHLSRGIGY	QQHYNGYT	0.001	
1-237	0.09		IGHV1-69*01	IGKV4-1*01	ARDSKAYDAFDI	LQYYSLPRT	0.001	
2-16	0.01	11	IGHV1-69*01	IGKV3-15*01	ARNVGAMEALGYLDV	QQYNHWPLS	0.001	
2-18	0.09	0.0009	IGHV3-72*01	IGKV1-12*01	ARGPHHSDRSGYYGGTFDI	QQGNMFPLT		0.004
2-24	6.4	0.04	IGHV1-69*01	IGKV1-33*01	ARAVFSYDSSADVVKPDTFDI	QQYDNLRWA		0.1
2-25		0.00009	IGHV1-3*01	IGLV3-1*01	ARDESTGDYYYYMDV	QAWDSDTYV		0.002
2-29	3.9		IGHV3-15*01	IGLV6-57*01	TTERRTSGYAAY	QSYDSTSQV		
2-32	0.005		IGHV4-59*01	IGKV4-1*01	ATAEHRIATPGS	QQYYLTPLA	0.0009	
2-33	0.009		IGHV3-66*02	IGKV3-20*01	VRQATGAFGM	QQYGNSPWT		
2-37	0.05		IGHV4-34*01	IGKV4-1*01	ARGLGWNSEGSDDAFDV	QQYYHIPLT		
2-43	0.003	15	IGHV6-1*01	IGKV4-1*01	ARGTLLVGPLAFDI	QQYYRIPYT	0.0007	
2-45	0.05	9.8	IGHV4-34*02	IGKV3-15*01	ARDDHPSYDYIWGTYRLDQGGIGY	QHYNNWPPWT	0.003	
2-46	0.004	0.03	IGHV4-b*02	IGKV1-33*01	ARSVWGGLRGYFDY	QHSDNLLFT	0.0009	
2-48	8.1	14.6	IGHV1-2*02	IGKV3-20*01	AKDIIPDGPWLQVT	QQYGSSPLWT	0.005	
2-52	2.5		IGHV4-34*01	IGLV1-51*01	ARVPPTRTRKSLRKYYYSFYYMDV	GTWDSSLSVV	4.8	
2-55	0.04		IGHV4-4*07	IGKV3-15*01	AREEGSSWYWYFDL	QQYDDWPYT		0.001
2-59	0.02	2.3	IGHV4-34*02	IGLV1-47*01	ARLLRDFDWVPRTYYFDY	ATWDDCLSAYV	0.0006	
2-65	5.7	6.4	IGHV1-2*02	IGKV3-20*01	AKDIIPDGPWLQVT	QQYGSSPRT	0.002	
2-111	0.03		IGHV3-33*01	IGKV3-11*01	ARDGDSGHSFDY	QQRSNWPLT	0.9	
3-7	0.07	0.08	IGHV1-18*01	IGKV1-27*01	ARDGYNWGFLDF	QKYNSAPLT		0.001
3-16	0.1	0.09	IGHV1-18*01	IGKV3D-15*01	ARDAENWGFFDD	QQYNTWPYT		0.0007
3-18	1.5		IGHV3-21*01	IGKV1D-8*01	ARDNVLLWFGELLSHQKYYYYYGMDV	QQYYSFPRT	0.05	
3-22	0.009		IGHV3-7*03	IGLV3-25*03	ASLTVVTMVGSIDDY	QSADSSGTYQVV		
3-25	0.04	0.3	IGHV3-30-3*01	IGKV3-11*01	AREGYCGDDRCYSGQPDY	QQRSHWPPLT	0.0005	
3-29	0.003		IGHV1-46*01	IGKV2-28*01	ALAATLDGFGH	MQGLQTPIT	0.2	
3-35	2.5		IGHV3-30*03	IGLV5-45*02	AKSDYYIHKPAVLWFREFQTKYGMDV	MIWHSSAYV	0.4	
3-38	0.06		IGHV1-18*01	IGKV4-1*01	ARDATLHDRRSSGH	QQYYSTPYT	0.0007	
3-54	0.4	1	IGHV1-46*01	IGKV1-5*03	ARDYRPLGYPDNRLIAPALFDP	LQPDDFATYYCQHYDSFSMYT	0.0004	
3-58	0.9		IGHV3-30*03	IGLV3-10*01	AKDLGPVNLGWASYDFWSGYSTHKGYYYYYGMDV	YSTDSSGNHRGV	3.7	
3-61	3		IGHV3-21*01	IGKV1D-8*01	ARLGGEPTYYDFWSGYYTRYTGYFDY	QQYYSFPYT		4
3-65	0.002		IGHV3-33*01	IGKV4-1*01	ARARAPYDSSGYFADY	HQYDRTPFT	0.0003	
3-74	0.2		IGHV3-30-3*01	IGLV3-25*03	ARVYDFWSGYYGWVRGGRDYYYYYGMDV	QSADSSGTYHV	0.01	
3-76	2.2	5.5	IGHV4-39*01	IGKV1D-8*01	ARLSRQGLYDFWSGLVRRDPPAVYWYFDL	QQYYSFPYT		0.05
3-93	1		IGHV3-30*03	IGKV4-1*01	AKGGRKRVLLWFREFPRQSHDWYFDL	QQYYSTPLFT	0.9	
3-98	6.5	12.4	IGHV1-2*02	IGLV1-44*01	ARSRDGGYHKTYYYHNMDV	AAWDDSLNGPV		0.2

Notes: The activities of 56 antibodies were analyzed in AD169rev binding ELISA and neutralization assays. Their activities are expressed as virus-specific EC_50_ or IC_50_, respectively, defined as the concentration of IgG needed to achieve 50% of maximal signal in binding to AD169rev virus in ELISA or blocking 50% viral entry in ARPE-19 cells. Antibody-titration ELISA was used to determine the relative binding affinity of each mAb to HCMV antigens (recombinant gB, and recombinant pentameric gH complex). The EC_50_ value was calculated using four-parameter curve fitting, representing the concentration of mAb to reach 50% of maximal binding signal (see Materials and Methods). If there is no activity, the cell is blank

The number of mAbs in each donor correlated with the donor’s NT_50_ titers (Table 1), but the correlation was not statistically significant (correlation coefficient (*r*) = 0.97, *p* = 0.157). Of the panel, nine, three, and two neutralizing mAbs were identified from donors 1, 2, and 3, respectively. There was no statistically significant correlation between the number of neutralizing mAbs and donor NT_50_ titer (correlation coefficient (*r*) = 0.97, *p* = 0.158), possibly due to the low number of neutralizing mAbs recovered from the positive hits. Since we only sampled memory B-cells enriched from 80 mL fresh blood, less than 2% of total blood volume in average adults, we could not rule out the possibility that the cells of isolated mAbs represented only a small portion of each host’s total HCMV-specific memory B-cells. Nonetheless, of the 23, 17, and 16 mAbs isolated from donors 1, 2, and 3, respectively, we observed unique heavy or light CDR3 sequences for each antibody, confirming that these mAbs were genetically distinct (Table 2).

### Antigen specificity of the identified antibodies

HCMV virions have four major glycoprotein complexes: gB, gM/gN, gH/gL/gO, and the pentameric complex of gH/gL/pUL128-131 linked to virus infection of endothelial and epithelial cells [36-38]. Given the availability of the gB and the pentameric complex as recombinant antigens, we evaluated antigen specificity for all mAbs in ELISA, and their relative binding affinities were calculated as antigen-specific EC_50_ values (Table 2). Thirty-two mAbs were specific for the gB with twelve, ten, and ten isolated from donors 1, 2, and 3 respectively. Nineteen were specific for the pentameric complex with ten, four, and five isolated from donors 1, 2, and 3 respectively. Five mAbs, one each from donors 1 and 3, and three from donor 2, had unknown antigen specificity.

**Figure 1:**
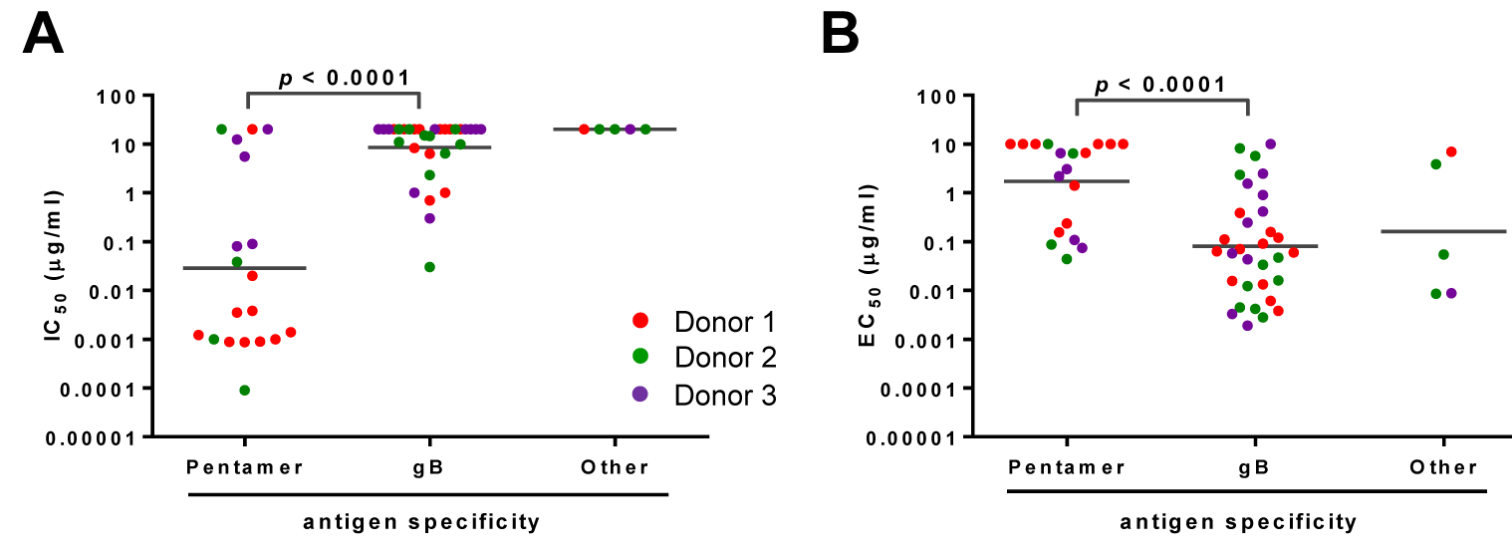
Functional attributes of the HCMV antibodies based on their antigen specificity The mAbs were grouped based on their antigen specificity for neutralizing potency (panel **A.)** and relative binding affinity to HCMV (panel **B.)** IC_50_ or virus-specific EC_50_ of antibodies are defined as the concentration of the IgG required to block 50% viral entry in ARPE-19 cells or to achieve 50% of the maximal binding signals in virus-specific ELISA. Antibodies from different donors are indicated by different colors. Nineteen mAbs reacted to recombinant “pentamer” and thirty-two to “gB.” The “other” represents mAbs not reactive to either gB or pentameric complex in ELISA. The lines represent the geometric means for the group. Unpaired two-tailed *t*-test was performed. Differences with statistical significance are shown with *p* values. The data are representative of two independent experiments.

To better understand antigen specificity and the correlation with function for each antibody, we plotted the antibodies’ IC_50_ and virus-specific binding EC_50_ values based on their antigen specificity. In epithelial cells, the pentamer-specific antibodies showed markedly greater neutralization potency compared to the gB-specific antibodies, with the geometric means of IC_50_ values at 0.03 *versus* 8.5 µg/mL (Figure 1A). An opposite trend was observed for virus-binding activity, as the virus-specific EC_50_ geometric means were 0.08 and 1.7 µg/mL for gB- and pentamer-specific antibodies, respectively (Figure 1B). Thus, among these mAbs, the potent neutralizing antibodies targeted the pentameric complex rather than gB, whereas the gB-specific antibodies showed higher binding affinity for whole virions. This result was consistent with the previous reports [27, 31].

**Figure 2:**
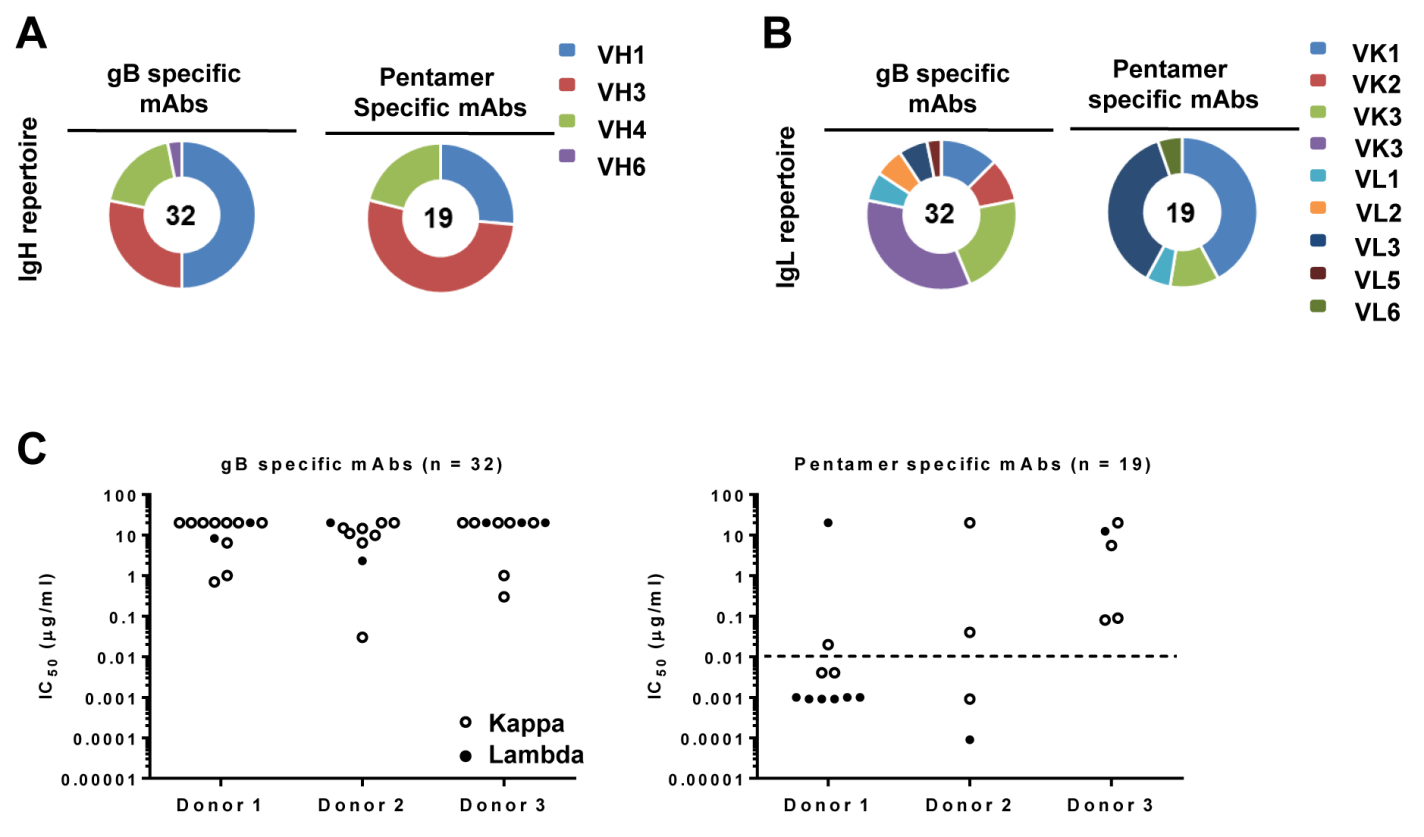
Heavy- and light-chain immunoglobulin gene usage Heavy-chain (panel **A.)** and light-chain (panel **B.)** family distributions of the 56 mAbs are shown according to their specificity for gB and pentamer. Numbers of antibodies analyzed are marked in the center of pie charts. Slices in the pie charts represent percentages of total heavy-chain or light-chain alleles. Light-chain usages (panel **C.)** are shown for gB-specific antibodies (left, *n* = 32) and pentamer-specific antibodies (right, *n* = 19). Closed circles indicate λ and open circles κ light-chain antibodies. Functional potency for each antibody is measured by IC_50_, which represents the IgG concentration needed to block 50% viral entry in ARPE-19 cells. The dashed line represents an IC_50_ value of 0.01 µg/mL, which is about 100 times more potent than HCMV-HIG.

In addition, the gB and pentamer-specific mAbs displayed differential preference for V_L_ gene usages (Figure 2A and 2B). The λ light chains seemed to be more frequently used by those pentamer-specific antibodies with IC_50_ values ≤0.01 µg/mL (Figure 2C, below the dotted line), as seven out of ten pentamer-specific mAbs from donors 1 and 2 contained λ light chains.

### Analysis of donor B-cell repertoires and HCMV-specific B-cells

Next-generation sequencing (NGS) is an important tool to study the genetic diversity and evolution of functional antibodies [39-42]. Here, we investigated the HCMV mAbs within the host memory B-cell repertoires using the unbiased repertoire analysis method described previously [43]. We constructed antibody libraries using total RNA from ∼20 million peripheral blood mononuclear cells (PBMCs). Reverse transcription (RT) was used to produce template cDNA for 5’-RACE PCR to amplify the entire variable regions of IgG genes. The V_H_, V_κ_, and V_λ_ libraries of each donor were sequenced on the Ion Torrent PGM platform [43], which yielded on average 5.9-6.5 million reads per library. The data were processed by the Antibodyomics 1.0 pipeline [44]. The quality assessment of NGS results for donor 1 was provided as an example (Figure S4). Overall, 80.3-95.6% of the sequences contained the complete V(D)J coding region and were subjected to an in-depth bioinformatics analysis (Supplementary Table 1).

**Figure 3:**
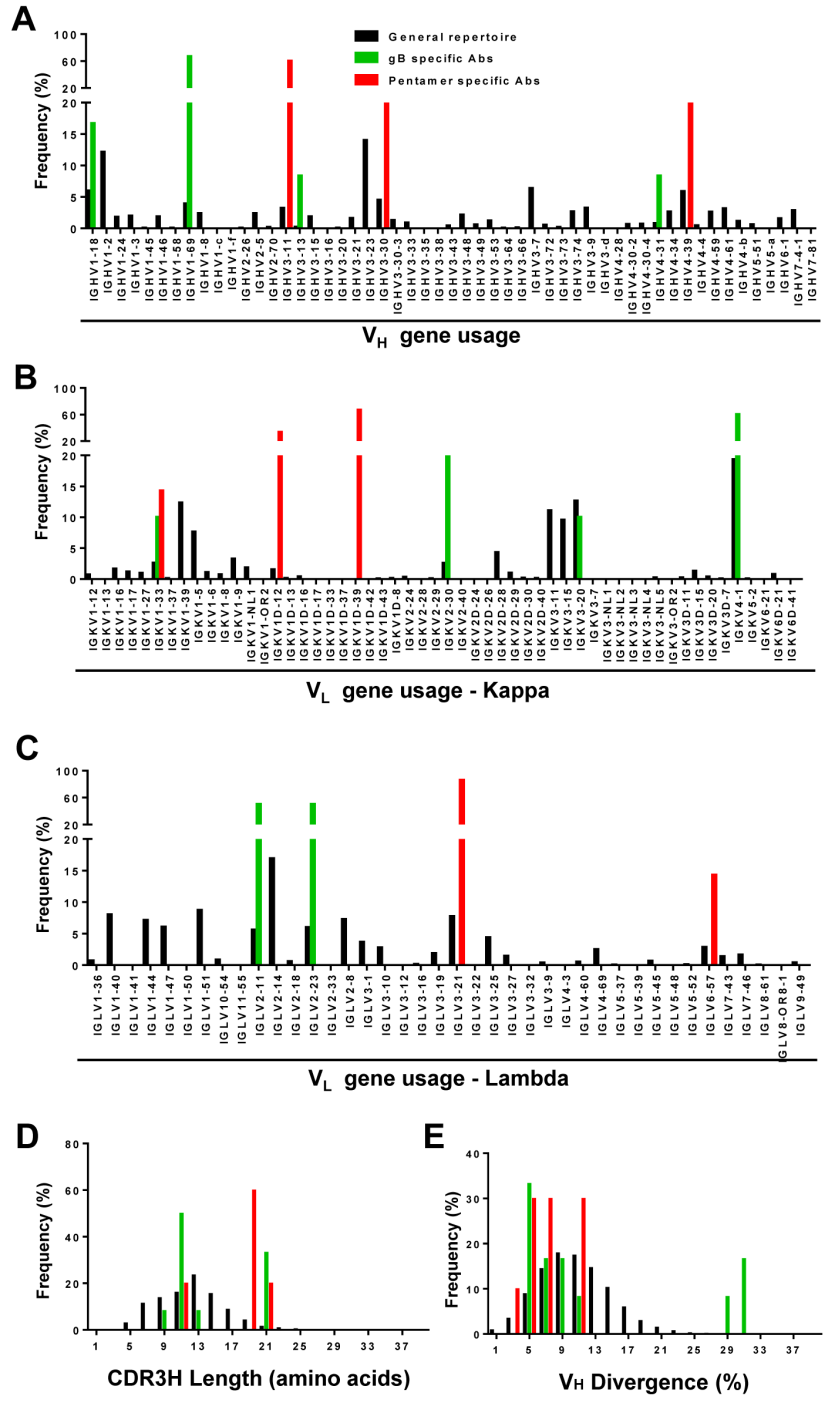
Repertoire distributions of antibodies in donor 1 Heavy- (V_H_) and light-chain (V_κ_ and V_λ_) repertoires were obtained from deep sequencing of libraries with 5’-RACE PCR amplification. The processed antibody (*n* = 23) chain sequences of NGS data (black), gB specific (green), and pentamer specific sequences (red) of isolated mAbs were used to calculate heavy- (panels **A, D, E)** and light-chain (panel **B, C)** repertoire properties such as germline gene usage for heavy-chain (panel **A)**, κ chain **B.**, and λ chain **C.**, complementarity determining region 3 length for heavy chain (CDR3H) **D.**, and V_H_ germline gene divergence **E.**

When plotted against the host B-cell repertoires, the distributions of gB- and pentamer-specific mAbs exhibited notable differences in germline gene usage, CDR3H length, and degree of somatic hypermutation (SHM). For heavy chain analysis of donor 1, IgHV1-2 and IgHV3-23 accounted for 12% and 14% of the total repertoire (Figure 3A). However, the majority of the pentamer-specific antibodies were derived from IgHV3-11, including mAbs 1-15, 1-64, 1-85, 1-125, 1-150 and 1-175 (Table 2). On the other hand, most gB-specific mAbs originated from IgHV1-69, including mAbs 1-155, 1-189, 1-190, 1-191, 1-192, 1-223, 1-224 and 1-237 (Table 2). For donors 2 and 3, IgHV4-39, and IgHV1-69 appeared to be the prevalent germline families, accounting for 13-15% and 6-7% of their respective V_H_ repertoires (Supplementary Figure 5). Similarly, four of the seventeen mAbs isolated from donor 2 were from the IgHV4-34 germline gene family (2-37, 2-45, 2-52 and 2-59) (Table 2), while five of the sixteen mAbs isolated from donor 3 were assigned to the IgHV3-30 germline gene (3-25, 3-35, 3-58, 3-74 and 3-93), with none of these mAbs derived from the most prevalent germline genes in their respective repertoires. For light chains, the three donors exhibited similar germline V gene usages (Figure 3B, 3C and Supplementary Figure S5), with IgKV4-1 being the most prevalent family in the V_κ_ repertoires. The pentamer-specific mAbs from donor 1 showed a preferred usage of V_λ_ germline genes (Table 2, Figure 3C), as described earlier for the potent neutralizing mAbs 1-15, 1-85, 1-125 and 1-175 (Figure 2C). Thus, our analyses demonstrated diverse, donor-specific germline usages in memory B-cell responses to HCMV. Moreover, generation of HCMV-specific antibodies appeared not to be associated with the frequency of germline genes in host repertoires. Although it may have led to the expansion of certain germline families, such as the six pentamer antibodies from IgHV3-11 in donor 1, latent HCMV infection in these donors had little effect on the overall host B-cell repertoires (Table 2).

We then compared CDR3H distributions determined from the HCMV-specific mAbs and from individual donor B-cell repertoires (Figure 3D, Supplementary Figure 5). The pentamer-specific mAbs appeared to possess longer CDR3H loops than both the gB-specific mAbs and the majority of antibodies in the general repertoires. However, due to small sample size, this difference was not statistically significant. We also calculated germline gene divergence to determine the degree of SHM. This calculation showed sparse distributions for the gB- and pentamer-specific antibodies in comparison to the general repertoires for the three donors (Figure 3E, Supplementary Figure 5). Taken together, our comparative analysis revealed a diverse pattern of germline gene usage for HCMV-specific antibodies among three donors, with no discernible patterns in CDR3H length or levels of SHM as compared to host B-cell repertoires.

### Complex B-cell lineage development for pentamer-specific mAbs

**Figure 4:**
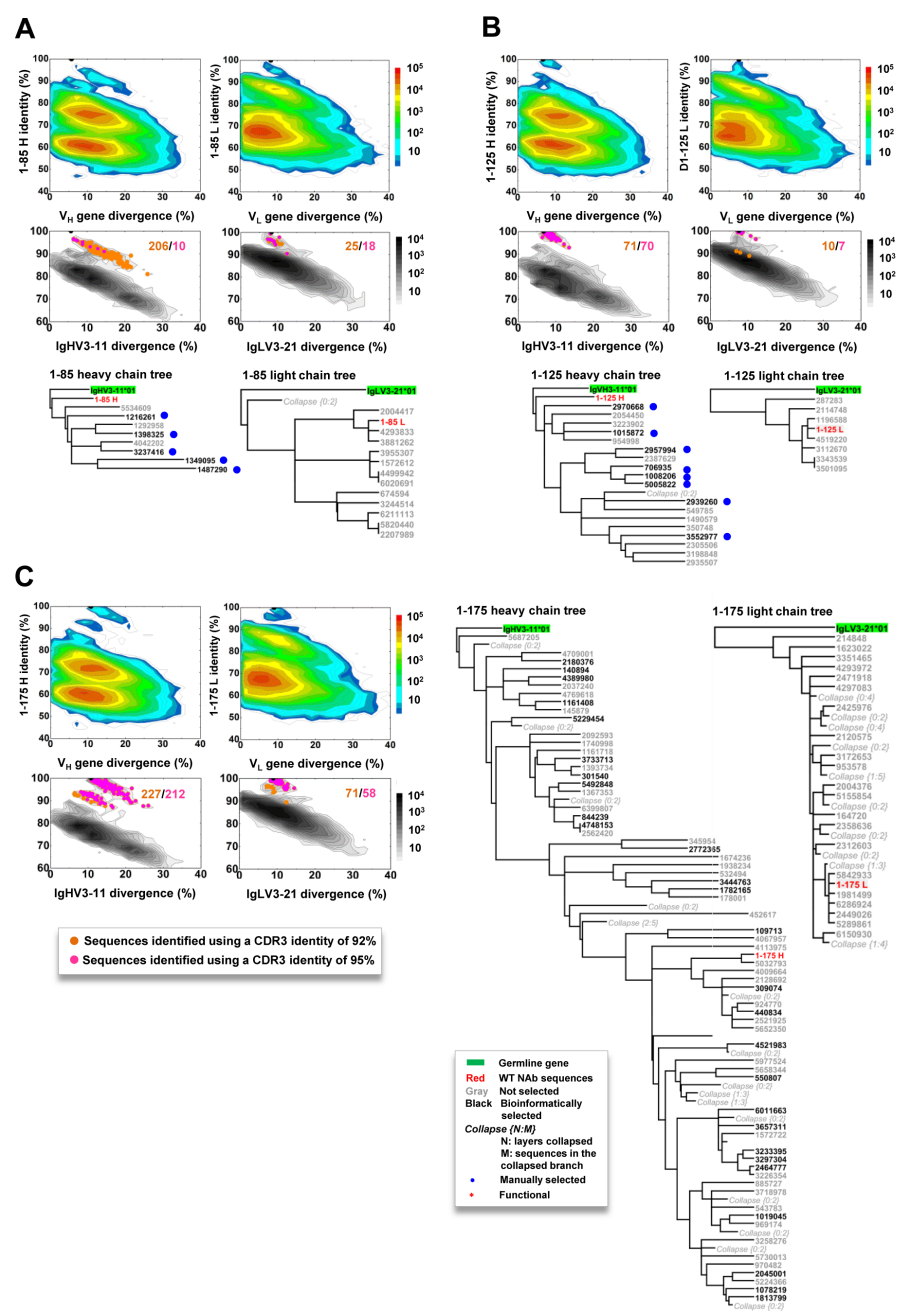
Lineage and correlation analysis of three pentamer-specific antibodies from donor 1 For mAbs 1-85 (panel **A)**, 1-125 **B.**, and 1-175 **C.**, an identity/divergence plot, CDR3-based lineage analysis, and phylogenetic analysis are combined to provide insights into lineage maturation and structure in the context of the repertoire (color coded) and the specific germline gene family that gives rise to the antibody (black contour lines). In the identity/divergence 2D analysis, sequences are plotted as a function of sequence identity to the template mAb and sequence divergence from putative germline genes. Color coding denotes sequence density. The maximum-likelihood tree of selected somatic variants with a CDR3 identity of 95% or greater to the template antibody is rooted by the putative germline gene and shown for both heavy and light chains.

Next, we probed the HCMV-specific mAb lineages in the context of donor B-cell repertoires by combining identity/divergence two-dimensional (2D) plots, CDR3-based lineage tracing, and phylogenetic analysis. We first applied the 2D analysis to identify somatic variants for all 56 mAbs. For the vast majority of the mAbs, sequence homologs were not identified, suggesting that somatic variants for these mAbs, if existent, were at a frequency below detectable levels within the general repertoire (Supplementary Figure S6). However, somatic variants could be recognized from the 2D plots for five pentamer-specific mAbs: three (1-85, 1-125, and 1-175) from donor 1 (Figure 4) and two (2-25 and 2-55) from donor 2 (Supplementary Figure S7).

For mAb 1-85, heavy- and light-chain somatic variants formed large islands on the 2D plot, extending from 100% identity towards the main population (Figure 4A). The extensive maturation in this lineage was evidenced by the presence of variants with a germline divergence of 5-10% greater than 1-85. When utilizing the 1-85 CDR3s to trace the lineage variants, we identified 206 heavy-chains and 25 light-chains with an identity of 92% or greater (Figure 4A). Thus, a large number of 1-85-like antibodies were present in the repertoire, with these heavy-chain variants more distant from the germline origin than 1-85 in the phylogenetic analysis (Figure 4A). For mAb 1-125, smaller somatic populations were observed on the 2D plots (Figure 4B). The CDR3-based lineage analysis, using an identity cutoff of 92%, yielded 71 and 10 variant sequences for heavy- and light-chains, respectively. All the heavy-chain variants of 1-125 were located on the more distant branches of the lineage tree rooted by their germline gene, IgHV3-11 (Figure 4B). For mAb 1-175, the 2D plots revealed a more complex pattern (Figure 4C). In the CDR3-based lineage analysis, 227 heavy-chains and 71 light-chains were found with CDR3 identity of 95% or greater when compared to that of 1-175. On the 2D plot, these heavy-chain variants formed two distinct islands. Phylogenetic analysis revealed that mAb 1-175 was sandwiched by less mutated parental branches and sequence groups with greater genetic distances to the germline gene. A similar but less complex branching pattern was observed for 1-175 light-chains. These analyses thus demonstrated varying degrees of lineage complexity for three pentamer-specific antibodies in donor 1. For the two mAbs from donor 2, we observed similar but less pronounced lineage diversity (Figure S7). It should be noted that, while mAbs 1-85, 1-125, 1-175 and 2-25 were potent in neutralization, mAb 2-55 only showed marginal neutralizing activity (Table 2). Thus, there appeared to be no correlation between the size and complexity of antibody lineages and their antiviral function. To summarize, our analyses revealed complex lineage development patterns for five pentamer-specific neutralizing antibodies from donors 1 and 2. However, such complex lineage patterns, characteristic of active B-cell evolution, were not observed for gB-specific or other pentamer-directed binding antibodies (Supplementary Figure S6), even within the same germline origin or the same donor.

To verify the lineage patterns revealed by the 5’-RACE PCR method, we conducted an additional deep-sequencing analysis using a single-molecule barcoding strategy and gene family-specific primers [43]. Multiple sets of primers were used to amplify IgG heavy-chains of the IgHV3-11 origin for mAbs 1-15, 1-85, 1-125, and 1-175 from donor 1, and of the IgHV1-3 origin for mAb 2-25 from donor 2 (Supplementary Table S2). The resulting heavy-chain libraries were sequenced and analyzed using the same methods. Interestingly, the gene-specific method captured somatic variants not only for 1-85, 1-125, and 1-175, but also for 1-15, which was not captured by the 5’-RACE PCR method, probably due to its low frequency in the general repertoire. Overall, the lineage patterns were similar to those observed in the 5’-RACE repertoire analysis (Supplementary Figure S8).

Taken together, our analysis demonstrated diverse, donor-specific B-cell repertoires that were inherent to the immune response to latent HCMV infection. The complex lineage structures of five pentamer-specific mAbs in donors 1 and 2 indicated an active maturation process, likely driven by repeated antigen encounters. Since both donors 1 and 2 were latently infected, the recall antigen exposure could have come from silent viral episodes of reactivation or super-infection with no clinical presentation of symptoms. In addition, the high NT_50_ titers in donors 1 and 2 could be a result of the active maturation of the pentamer-specific antibodies. In contrast, donor 3 with the lower NT_50_ titer did not exhibit such a complex lineage development pattern. Intriguingly, the silent viral episodes seemed to have limited effects on gB-specific memory B-cells, as no gB antibody from any donor displayed patterns of extended lineage development (Supplementary Figure S6).

### Functional validation of NGS-derived antibody somatic variants

**Figure 5:**
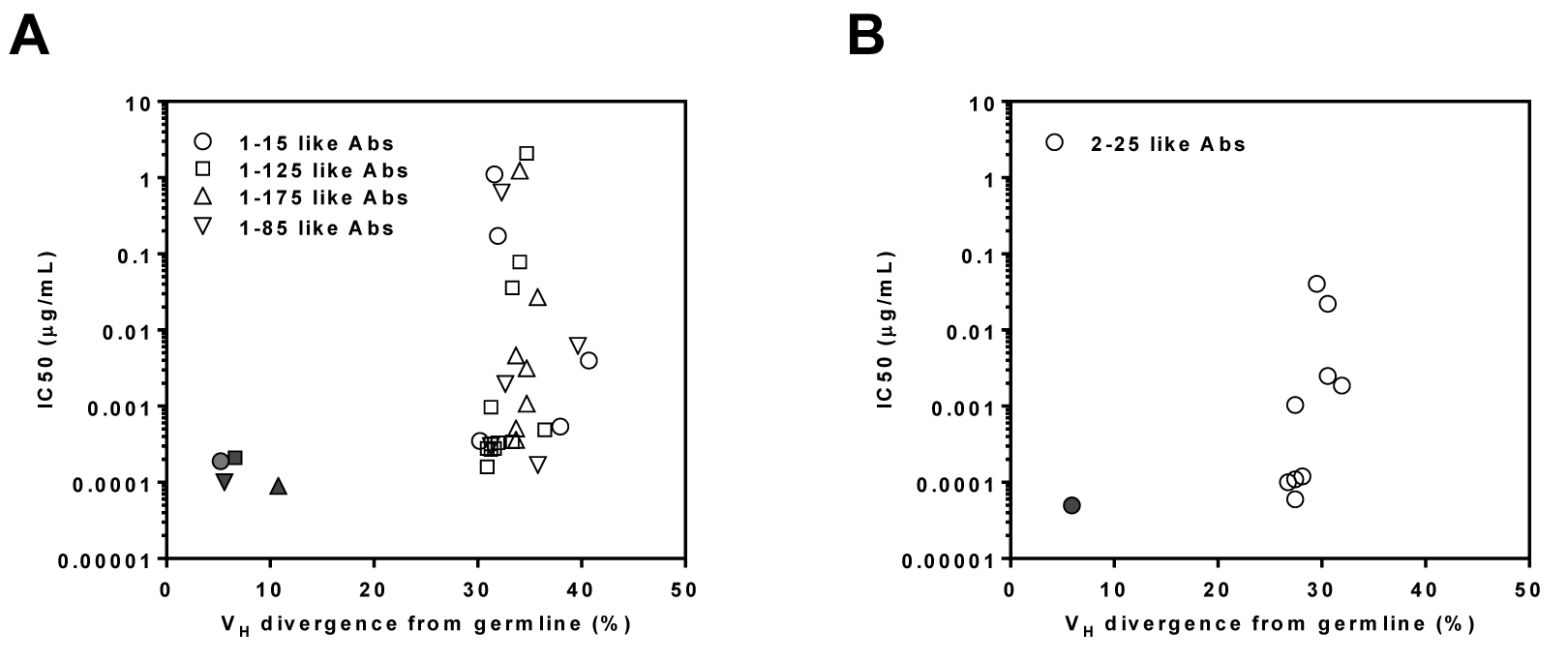
Antiviral function of reconstituted somatic variant antibodies mAbs 1-15 like (circle, *n* = 6), 1-125 like (square, *n* = 13), 1-175 like (regular triangle, *n* = 8) and 1-85 like (inverted triangle, *n* = 6) antibodies of donor 1 **A.** and 2-25 like antibody (circle, *n* = 10) of donor 2 **B.** were paired with their respective parental reference antibody light-chains and expressed as reconstituted antibodies. The reconstituted antibodies were then tested for neutralizing activity in ARPE-19 cells. For each reconstituted antibody, based on their lineage group, a plot is used to assess correlation between their IC_50_ (*y*-axis) and V_H_ divergence from germline (*x*-axis) of antibodies. Solid symbols refer to the correlation of the parental reference antibodies. The data are representative of two independent experiments.

To verify NGS-derived somatic variants, we selected a total of twenty-nine IgHV3-11-originated heavy-chains from the lineages of mAbs 1-15, 1-125, 1-85, and 1-175, either from the 5’-RACE repertoire analysis or from the gene-specific antibody family analysis. After pairing with the respective light-chains from the reference mAbs 1-15, 1-125, 1-85, and 1-175, the reconstituted antibodies were tested in neutralization assays. The IC_50_ value was then plotted against each somatic variant’s germline gene divergence (Figure 5A). Similarly for mAb 2-25, three heavy-chains from the 5’-RACE repertoire and six from the gene-specific methods were paired with the 2-25 light-chain. The reconstituted antibodies were analyzed for neutralizing potency in correlation to their V_H_ divergences (Figure 5B). The results indicated that the majority of reconstituted mAbs displayed lower neutralizing activity than their parental reference antibodies. Since the light-chain CDRs could contribute to the overall function of a given mAb, one possible explanation was that non-native pairing of NGS-derived heavy-chain variants with the reference light chain might have affected the neutralizing activity. Nevertheless, the neutralization data supported our hypothesis that most of the NGS-derived heavy-chains within these lineages were functional, reflecting active antibody maturation in response to repeated exposures to the recall antigen.

## DISCUSSION

To better understand antiviral humoral immunity in the context of natural infection, we isolated 56 antibodies from HCMV-specific memory B-cells in three donors with a wide range of NT_50_ titers. Consistent with previous reports [27-29, 31, 45, 46], potent neutralizing mAbs targeted the pentameric complex as opposed to gB. By probing the host B-cell repertoires with CDR3 signature sequences of each mAb, we observed distinct patterns of extended B-cell lineage development for five mAbs to the pentameric complex in two of the three donors. Since memory B-cells can accumulate somatic mutations upon antigen encounters, somatic variants in the repertoire divergent from these five mAbs would be indicative of pentamer-specific antibody maturation, driven by repeated antigen encounters. In contrast, such maturation patterns were not observed for any gB-specific antibodies (Supplementary Figure S6). Our results provided the first genetic evidence that active B-cell lineage development can occur in healthy donors with latent HCMV infection, and more importantly, such extended maturation pattern was limited to antibodies specific to the pentameric complex. This finding was consistent with the knowledge that the pentameric complex is a target accounted for the majority of neutralizing activities in HCMV hyper-immune globulin [25, 26] and also the target for mAbs potent in viral neutralization [27-29, 31, 45]. Consequently, such genetic evidence of active maturation of the pentamer-specific antibodies in donors 1 and 2 was correlated with their high neutralizing titers, which was not observed for donor 3. Furthermore, the result that memory B-cells specific to the pentamer have undergone more active and diverse lineage development than the memory B-cells to gB suggested that antibodies to the pentamer, in contrast to those to gB, could play a functional role in response to recurrent infections during latency.

Recurrent HCMV infections in healthy seropositive subjects rarely present any clinical symptoms. Such largely silent viral episodes can only be captured by detection of viremia or viral shedding in urine or saliva of study subjects [3, 47]. Moreover, there could be minor viral episodes only with localized pathology in vascular endothelial cells or epithelial cells in solid organs such as kidney or lung, which may not lead to any viremia or viral shedding. However, the host immune system, evolved as a sentinel for foreign antigens, could “record” such antigen encounters. Involvement of host T-cells has been recognized as such an immune mechanism. and memory T-cell inflation is likely caused by frequent antigen encounters from silent sub-clinical viral events [20]. Contrary to memory T-cells, memory B-cells can archive such antigen encounters within their repertoire by accumulating somatic mutations. Thus, the pentamer-specific antibody lineage patterns identified in donors 1 and 2 could be viewed as indelible records of sub-clinical episodes in these two subjects. However, our current data would not be able to explain why such extended lineage pattern was not observed for antibodies from donor 3. Another observation that remains to be explained is the evidence that memory B-cells specific to gB were not activated with recurrent viral episodes in donors 1 and 2. A prospective case-control study using some of the technologies presented in this study could shed light on these fundamental questions related to sub-clinical HCMV recurrent infections in healthy subjects.

It should be noted that not all pentamer-specific antibodies showed similar degrees of lineage evolution. For example, mAbs 1-15, 1-64, and 1-150 from the IGHV3-11 germline exhibited no detectable maturation as compared to mAbs 1-85, 1-125 and 1-175 of the same germline in donor 1 (Supplementary Figure S6A). These differential patterns in lineage development were unlikely due to epitope specificity, since most of these mAbs of the IGHV3-11 germline in donor 1 can cross-compete with each other [48]. A plausible explanation might be that the rare memory B-cells in the repertoires were presented at very low frequencies in the library constructed by 5’-RACE PCR amplification. Thus, their genetic variants were below the detection level of the current deep sequencing methods. Indeed, once gene family-specific primers were used for amplification, we were able to capture the somatic variants of mAb 1-15, which displayed similar degrees of lineage maturation when compared to that of mAb 1-85 (Supplementary Figure S8).

Of note, none of the NGS-derived somatic variants tested in this study showed improvement in potency over their parental reference mAbs (Figure 5). This result differed from the previous reports on HIV-1 broad neutralizing antibodies (bNAbs), where the NGS-derived variants often demonstrated greater neutralization breadth and/or potency than the experimentally identified bNAbs [40, 42, 49]. Besides the potential impact of non-native pairing of the heavy and light chains, the difference in our study could also be attributed to differential effects of viral pathogenesis on the host B-cell repertoires. Chronic HIV-1 infection is associated with a high rate of viral mutation [50] and elite bNAbs are rare species among antibodies to HIV-1. These bNAbs would have to survive continuous selection for both heavy- and light-chain fitness in the midst of massive B-cell lineage expansion and maturation [51, 52]. On the contrary, the genes encoding the pentameric complex are largely conserved [48, 53], and recurrent HCMV infection during the latent phase is likely of local lesions occurring only periodically. The B-cells activated by HCMV, although capable of evolving, were not under the same level of selection pressure as those driven by chronic HIV-1 infection.

Lastly, since we could not isolate any virus which was latent in these donors, we would not be able to test whether the antibodies characterized in this study had restricted specificity to the host strain. The virus used in our screening and viral characterizations was derived from AD169, a strain widely used in viral assays in the field, and immune sera from over 300 HCMV seropositive donors can neutralize this virus [24]. In addition, our recent report suggests that mAbs targeting the conserved regions of the pentameric complex have broad coverage against a collection of 11 clinical viral isolates [48]. Thus, the risk of missing potential hits using the AD169 strain for screening was low.

In summary, we have taken a systematic approach to characterize HCMV-specific B-cell responses in healthy individuals with latent infection. By combining isolation of mAbs and host B-cell repertoire analysis, we demonstrated extended lineage development patterns for memory B-cells producing antibodies to the viral pentameric complex, but not for those directed to gB. The results indicated that memory B-cells with antiviral function such as neutralization could be frequently mobilized, probably responding to silent viral episodes in the host. , although it was difficult to link the B-cell evolution to specific viral episodes due to the “silent” nature of HCMV recurrent infection in healthy subjects. Nonetheless, this study suggests that neutralizing antibodies could play an important role in control of recurrent HCMV infection in healthy subjects.

## MATERIALS AND METHODS

### Cells, recombinant proteins and viruses

Recombinant gB protein was designed based on the sequence of Towne strain with its furin-cleavage site mutated and the transmembrane region deleted. The protein was then expressed in HEK293 cells (Invitrogen) by transient transfection [54] (Sino Biologicals Inc.). Recombinant pentameric gH complex was produced in CHO cells (ATCC) as previously reported [25, 26]. Revertant AD169 virus (AD169rev virus) was propagated in APRE-19 cells (ATCC) as described previously [33, 34].

### Ethics statement

The study protocol was reviewed and approved by the internal ethical committee of the Research Laboratories of Merck & Co., Inc., Kenilworth, NJ, USA. The healthy adult volunteers with natural HCMV infection were recruited with written informed consent. The blood sampling was within the established NIH guidelines. Serum HCMV neutralization titers (NT_50_) for the three subjects at the time of sampling were determined as described previously [34].

### Ion torrent PGM sequencing of human antibody libraries, and bioinformatics analysis of antibody sequencing data

The 5’-RACE PCR protocol for unbiased antibody repertoire capture was modified as previously described [43]. The Antibodyomics 1.0 pipeline was used to analyze the HCMV antibody repertoires [40, 42, 43]. The modified protocols are detailed in Supplemental Experimental Procedures.

### Statistical analysis

Geometric means with 95% confidence intervals and *t*-tests were conducted using the GraphPad Prism^®^ 5 software. The linear regression correlation analysis was performed using GraphPad InStat^®^ 3.

## SUPPLEMENTARY MATERIALS FIGURES AND TABLES


