# Supplementary material for: Active evolution of memory B-cells specific to viral gH/gL/pUL128/130/131 pentameric complex in healthy subjects with silent human cytomegalovirus infection
Source: Oncotarget. 2017 Jun 3;8(43):73654–69. doi: 10.18632/oncotarget.18359 (PMC5650289; doi:10.18632/oncotarget.18359)
Supplement: Supplementary file 1 [file oncotarget-08-73654-s001.pdf]

Active evolution of memory B-cells specific to viral gH/gL/pUL128/130/131 pentameric complex in healthy subjects with silent human cytomegalovirus infection

SUPPLEMENTARY MATERIALS

Supplementary Table 1: Unbiased antibody repertoire analysis of three donors

Donor	PGM chip	Raw read (N)	After assignment (N)	Ig Chain	Read per Ig Chain (N)	Average length (nt)	Percent usable (%)
Dn1	318 v2	6,467,005	3,991,933	H	1,388,527	528.4	88.2
				κ	1,456,992	492.2	80.8
				λ	1,146,414	514.5	86.3
Dn2	318 v2	6,058,023	3,613,511	H	1,381,976	529.8	89.7
				κ	1,135,544	494.8	80.3
				λ	1,095,991	511.4	86.3
Dn3	318 v2	5,684,630	4,743,611	H	1,732,843	574.0	95.6
				κ	1,650,977	545.2	92.2
				λ	1,359,791	556.0	94.3

Note: The antibody repertoire analysis was performed using a human 5’-RACE PCR procedure for library preparation and long-read sequencing on the Ion Torrent PGM platform. Listed items include donor designation, PGM sequencing chip, total number of raw reads, number of remaining reads after germline gene assignment with a cutoff E-value of 10^{-3}, antibody chain type (H, κ, and λ), number of read per antibody chains average read length in nucleotides, and percent of usable sequences after *Antibodyomics1.0* pipeline processing.

Supplementary Table 2: Gene-specific NGS analysis of selected neutralizing antibody lineages from donors 1 and 2.[1]

Donor	PGM chip	Germline	Barcoded reads (%)	Read (N)	Average length (nt)	Usable reads after assignment (N)
Dn1	314 v2	IgHV3[2]	82.7	642,237	410.1	65,751
Dn2	316 v2	IgHV1[3]	90.4	1,206,836	428.4	244,644
	314 v2	IgHV1[4]	72.3	260,710	306.5	7,856

[1]The antibody libraries were prepared using gene-specific primers and single-molecule barcoding. NGS was performed on the Ion Torrent PGM platform. Listed items include the donor name, PGM sequencing chip, target germline gene, percentage of reads with correct 10-nt barcode length, total number of raw reads, average read length, and total number of sequences with assigned germline gene of IgHV3-11 for Dn1 and IgHV1-3 for Dn2 and with correct barcode length (10-nt) after *Antibodyomics 1.0* pipeline processing.

[2]The primers used here included those derived from unbiased repertoire NGS (UP) and from framework1 (FP):
1-15_FP: CAGGTGCAGCTGGTGGCGTCT
1-85_UP: ATTATAAAAGGTGCCCAGTGT
1-85_FP: CAGGTGCACCTGGTGGAGTCT
1-125_UP1: ATAATAAAAGGTGTCCAGTGT
1-125_UP2: ATAATAAAAGGTGTCGAGTGT
1-125_FP: CAGGTGCAGCTGGTGGAATCT
1-150_FP: CAGGTGCAGCTGGTGGAGTCT
1-175_UP1: ATTATAAAGGGTGTCCAGTGT
1-175_UP2: ATTATAAAAGGTGTCCAGTGT

[3]The primers used here included generic VH1 primer (G) and those derived from unbiased repertoire NGS (UP):
VH1_GP: ACAGGTGCCCACTCCCAGGTGCAG
2-25_UP1: GCAGCCACAGGTGCCCGCTCC
2-25_UP2: GCAGCCACGGGTGCCCGCTCC
2-25_UP3: GCAGCCACAGGTAGCCCGGCTCC
2-25_UP4: GCAGCCACATGTAGCCCGGCTCC

[4]The primers used here included those derived from unbiased repertoire NGS (UP) and from framework1 (FP):
2-25_UP1: GCAGCCACAGGTGCCCGCTCC
2-25_UP2: GCAGCCACGGGTGCCCGCTCC
2-25_UP3: GCAGCCACAGGTAGCCCGGCTCC
2-25_UP4: GCAGCCACATGTAGCCCGGCTCC
2-25_FP: CAAGTGCAGCTCGTGGAGTCT

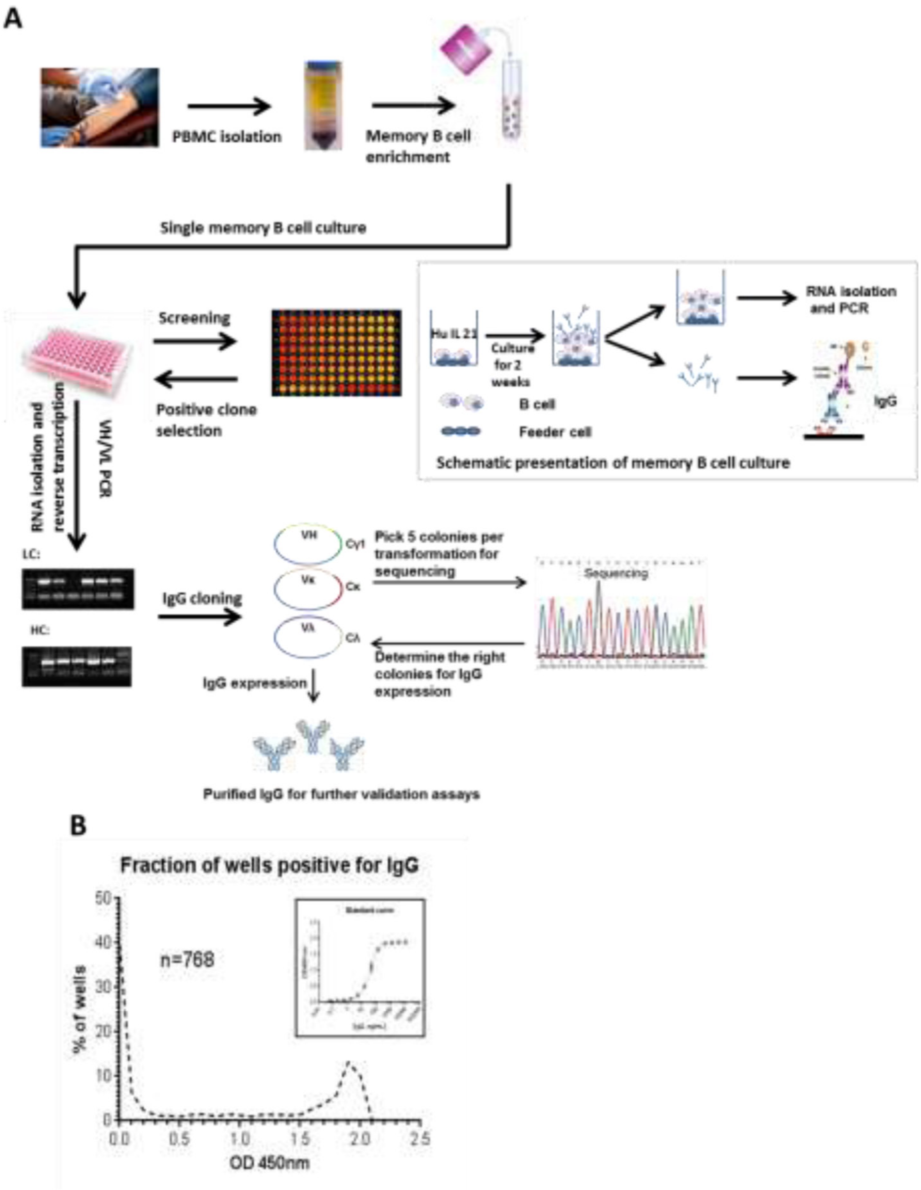

Supplementary Figure 1: Method outline of single memory B-cell culturing and IgG gene cloning (A) Flowchart for B-cell isolation and short-term culture to identify HCMV-specific antibodies. The enriched human memory B-cells were seeded at an average of 1.44 B-cells/well in 96-well U-bottom plates in the presence of 5,000 rad gamma irradiated 293 cells expressing human CD40 ligand at 40,000 cells/well and recombinant human IL-21 at 50ng/ml for 14 days. B-cell culture supernatants were then collected and tested for binding to HCMV virions and neutralization. Reverse transcription and cloning of IgG encoding genes were carried out using RNA from cell lysate of positive wells. Matched heavy chain and light chain genes were co-transfected into HEK293 cells and antibodies in the supernatants were purified for validation. (**B**) Fraction of IgG positive wells after short-term B-cell culturing. The supernatants from 8 96-well plates were randomly selected and assessed in human IgG ELISA as described in Supplementary Materials and Methods. OD 450nm values are plotted on *x*-axis against percentage of wells on *y*-axis. The insert shows the standard curve with purified human IgG.

A

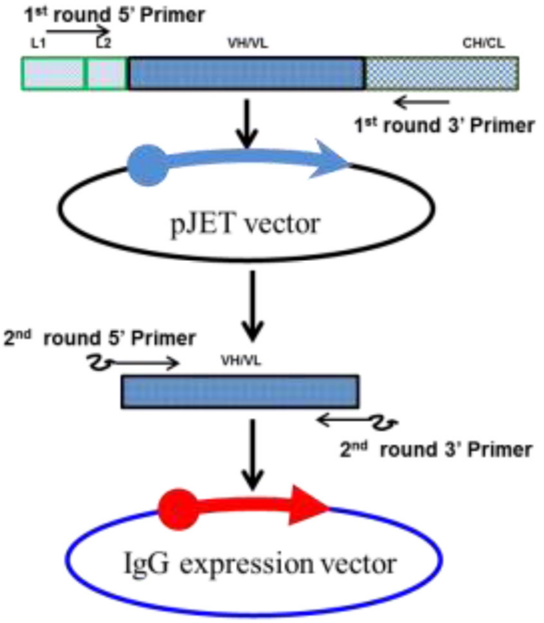

B

Primer name	Sequence 5' to 3'
HuLDRIGHV1	GGATCCTCTTCTTGGTGGCAGC
HuLDRIGHV3A	CTGAGCTGGGTTTTCCTYGTTG
HuLDRIGHV3B	CTATTTTARAAGGTGTCCAGTG
HuLDRIGHV4	AGCTCCCAGATGGGTCCTGTCC
HuLDRIGHV2	CATCCCTTCATGGGTCTTGTCC
HuLDRIGHV5	GGCTGTTCTCCAAGGAGTCTG
HuLDRIGHV6	CCTCCCATGGGGTGTCCTGTCA
HuLDRIGVk 1/2	ATGAGGSTCCCYGCTCAGCTGCTGG
HuLDRIGVk 3	CTCTTCCTCCTGCTACTCTGGCTCC
HuLDRIGVk 4	ATTTCTCTGTTGCTCTGGATCTCTG
HuLDRIGVλ 1	GGTCCTGGGCCCAGTCTGTGCTG
HuLDRIGVλ 2	GGTCCTGGGCCCAGTCTGCCCTG
HuLDRIGVλ3	GCTCTGTGACCTCCTATGAGCTG
HuLDRIGVλ 4/5	GGTCTCTCTCSCAGCYTGTGCTG
HuLDRIGVλ 6	GTTCTTGGGCCAATTTTATGCTG
HuLDRIGVλ 7	GGTCCAATTCYCAGGCTGTGGTG
HuLDRIGVλ 8	GAGTGGATTCTCAGACTGTGGTG
HuCH-R	GAAGACCGATGGGCCCTTGG
HuCλ-R	CACCAGTGTGGCCTTGTTGGCTTG
HuCk-R	GTTTCTCGTAGTCTGCTTTGCTCA

K=G+T, M=A+C, R=A+G, S=G+C, W=A+T, Y=C+T.

Supplementary Figure 2: Schematic drawing on cloning strategy and sequences of PCR primers used for initial amplification. (**A**) Schematic presentation of IG gene cloning strategy. (**B**) IG variable region genes were cloned using first round PCR primers which sequences are derived the leader region (5') and constant region (3') of human IgG genes. Products from the first round PCR were then cloned into the pJET cloning vector. Second round PCR primers were designed based on the sequences at the beginning of framework 1 and ending of framework 4 in pJET vector. A 15bp nucleotide with overlapping sequences with IgG expression vector is added into the second round PCR primers to facilitate downstream in-fusion cloning. IG variable region genes amplified from second round PCR were then cloned into IgG expression vector via in-fusion cloning.

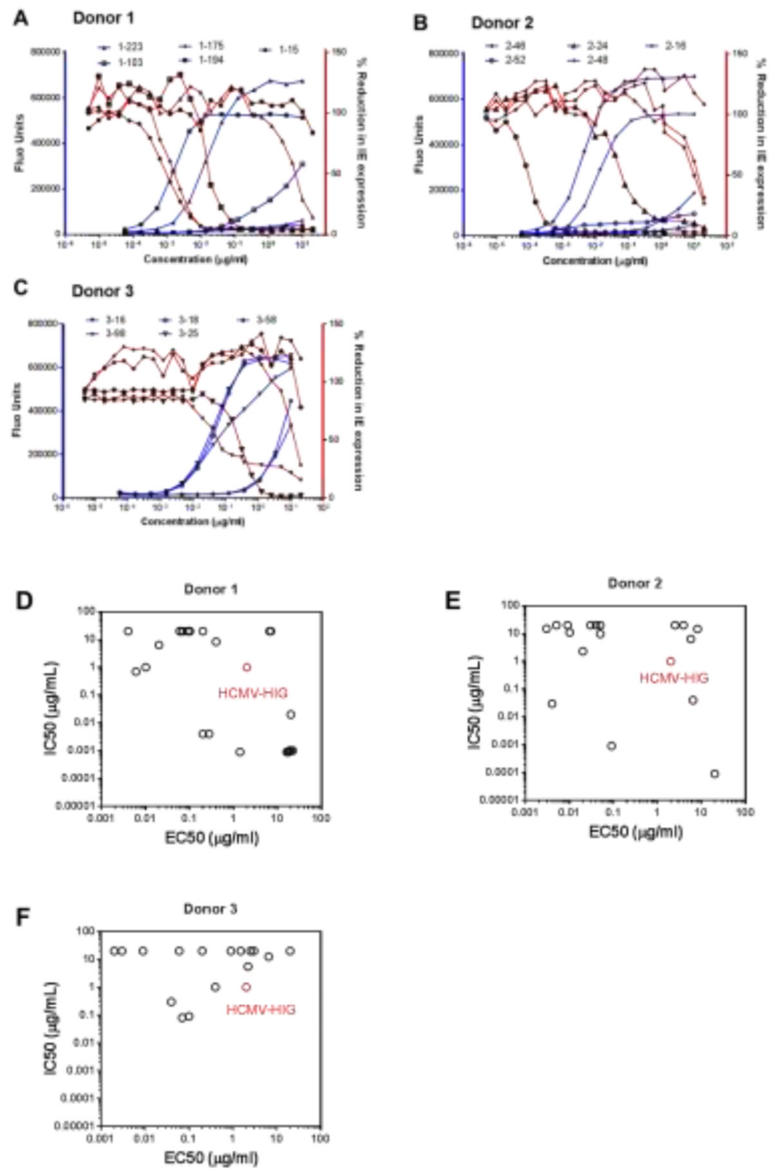

Supplementary Figure 3: Neutralizing and binding activities of representative antibodies. Antibodies from donor 1 (**A**), donor 2 (**B**) and donor 3 (**C**) were plotted for titration, with left *y*-axis (blue) shows HCMV virion binding activity, and right *y*-axis (red) indicates the neutralizing activity. Binding and neutralizing activities were tested as described in Supplementary Materials and Methods. The curves for binding and neutralizing activities are shown in blue and red, respectively. Correlation analysis of neutralizing and binding properties of 56 monoclonal antibodies from donor 1 (**D**), donor 2 (E) and donor 3 (F), with each mAb are plotted for its IC_{50} (*y*-axis) and virus-specific EC_{50} (*x*-axis) activities. The mAbs with virus-specific EC_{50} of > 20μg/ml are designated as no detectable binding activity and arbitrary values of 10 are assigned for plotting. The mAbs with IC_{50} of > 20μg/ml are designated as no detectable neutralizing activity and arbitrary values of 20 are assigned for plotting. The red open circle represents the control HCMV-HIG (CytoGam®).

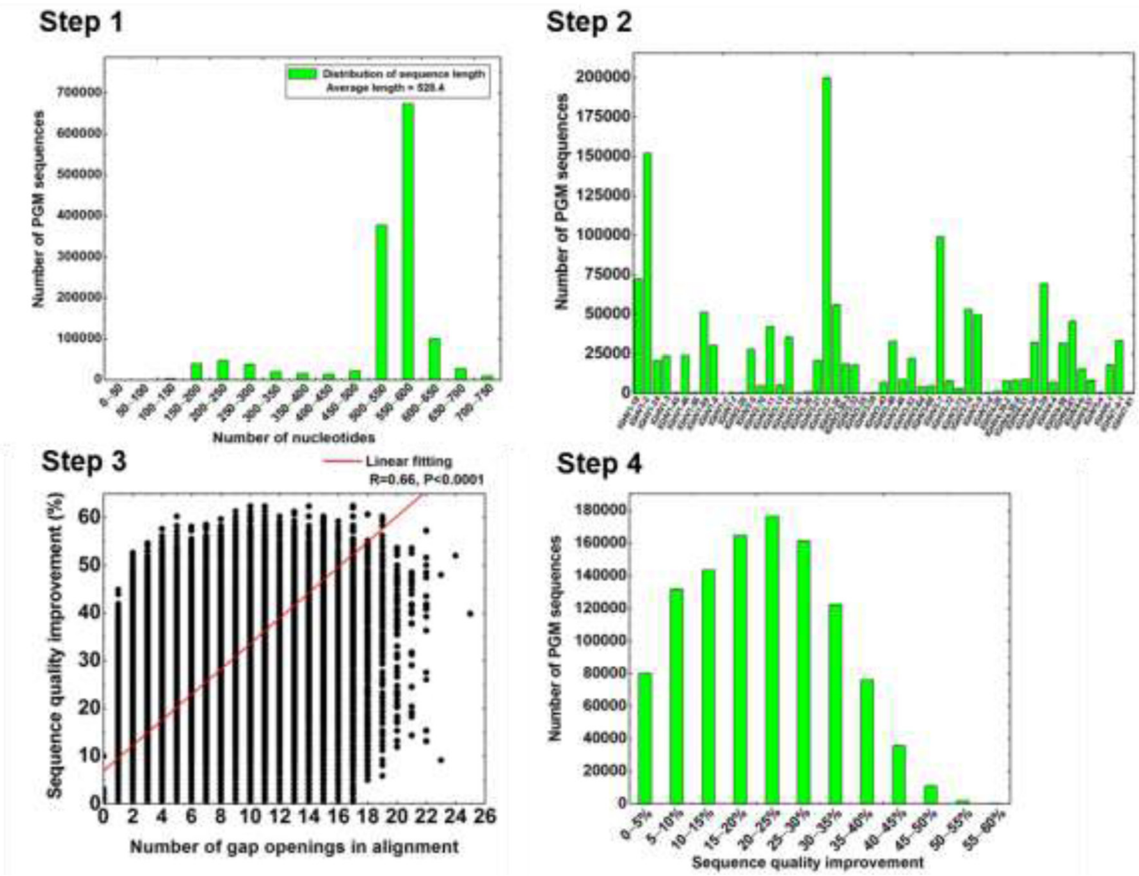

Supplementary Figure 4A: Pipeline processing of HCMV donor 1 (Dn1) heavy chain PGM sequencing data set. Read length distribution (step 1). Of 1,388,527 reads, 1,221,842 (or 88.3%) are over 400bp. Germline gene distribution (step 2). Correlation between number of gaps in V gene alignment and sequence quality improvement measured by the increase of sequence identity to germline gene after correction (step 3). The correlation coefficient is 0.66 and P-value is less than 0.0001. Distribution of sequence quality improvement (step 4), with an average of 20.9%.

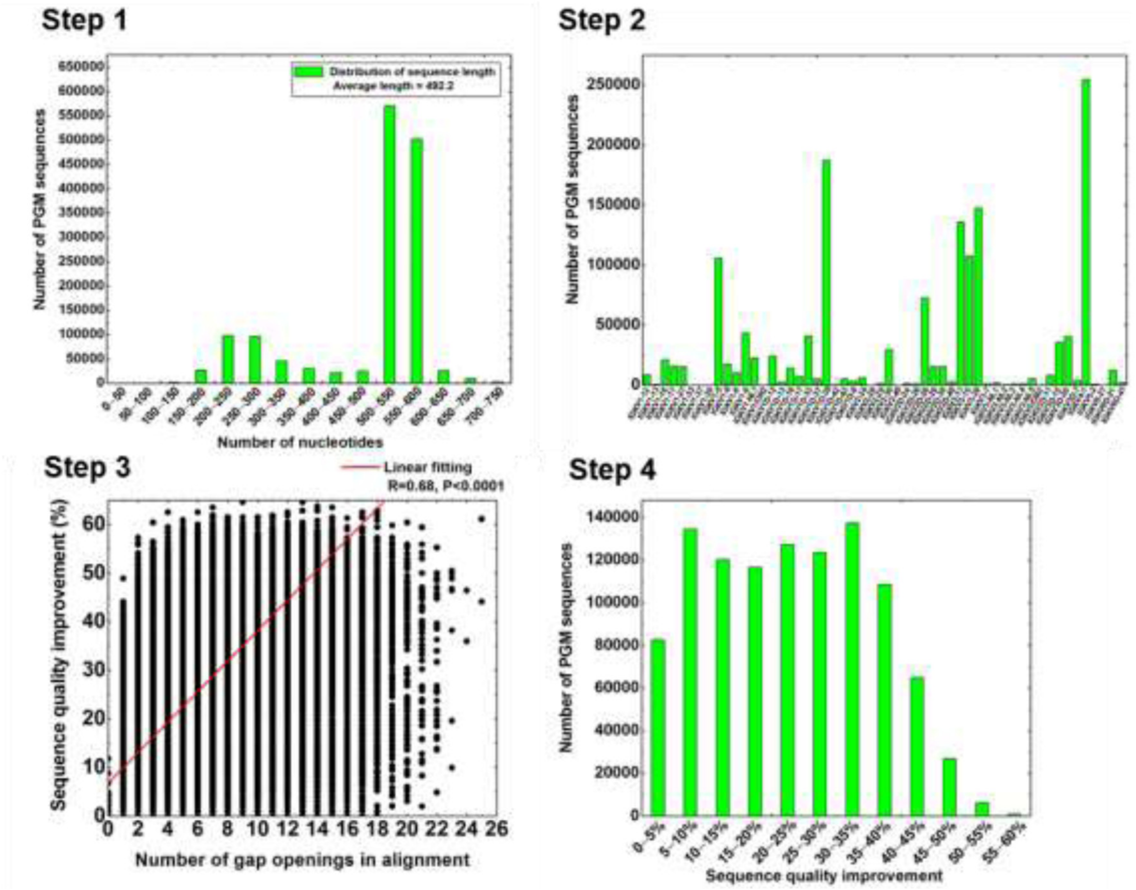

Supplementary Figure 4B: Pipeline processing of HCMV donor 1 (Dn1) Kappa chain PGM sequencing data set. Read length distribution (step 1). Of 1,456,992 reads, 1,156,480 (or 79.5%) are over 400bp. Germline gene distribution (step 2). Correlation between number of gaps in V gene alignment and sequence quality improvement measured by the increase of sequence identity to germline gene after correction (step 3). The correlation coefficient is 0.68 and P-value is less than 0.0001. Distribution of sequence quality improvement (step 4), with an average of 22.8%.

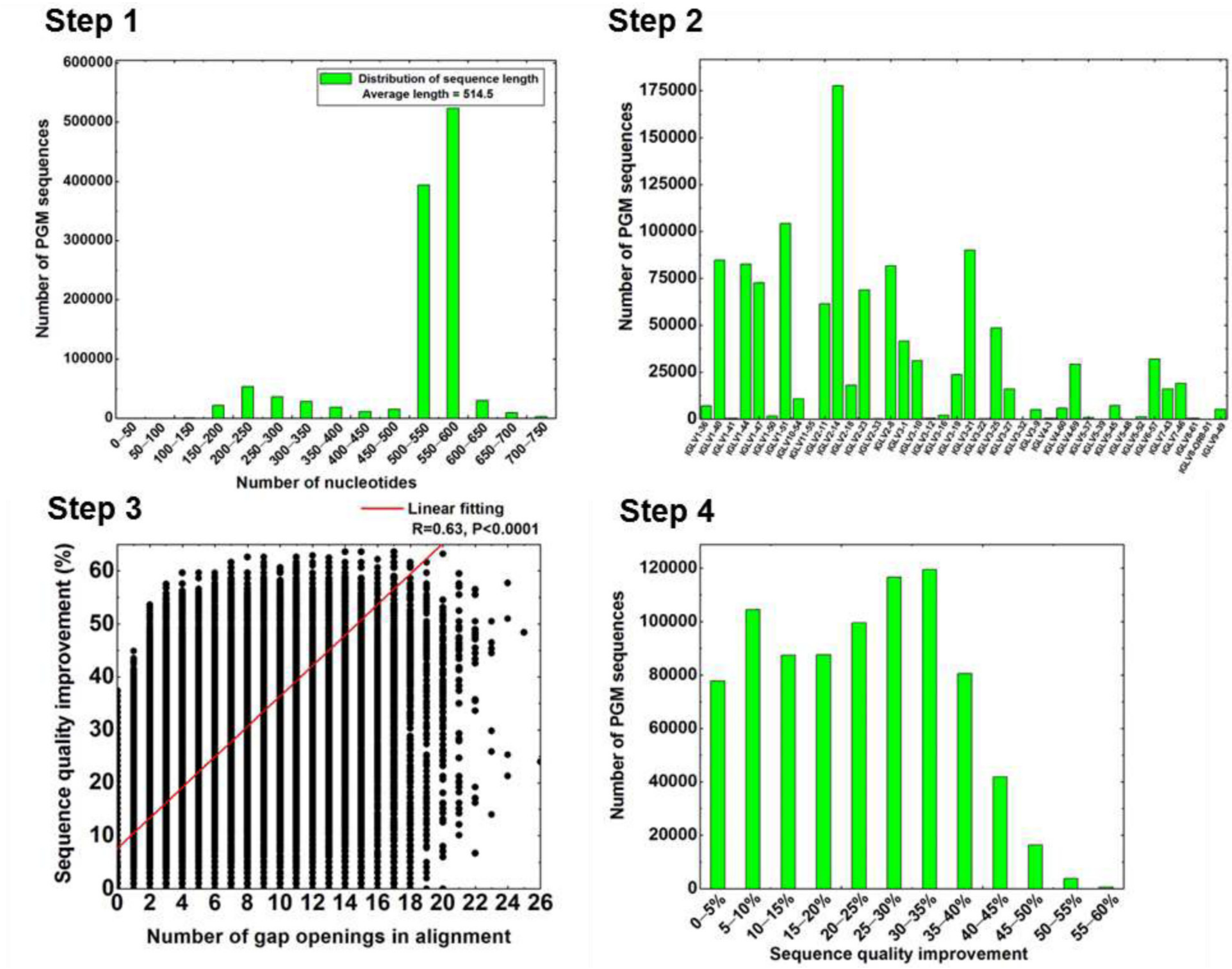

Supplementary Figure 4C: Pipeline processing of HCMV donor 1 (Dn1) lambda chain PGM sequencing data set. Read length distribution (step 1). Of 1,146,414 reads, 985,300 (or 86.0%) are over 400bp. Germline gene distribution (step 2). Correlation between number of gaps in V gene alignment and sequence quality improvement measured by the increase of sequence identity to germline gene after correction (step 3). The correlation coefficient is 0.63 and P-value is less than 0.0001. Distribution of sequence quality improvement (step 4), with an average of 22.3%.

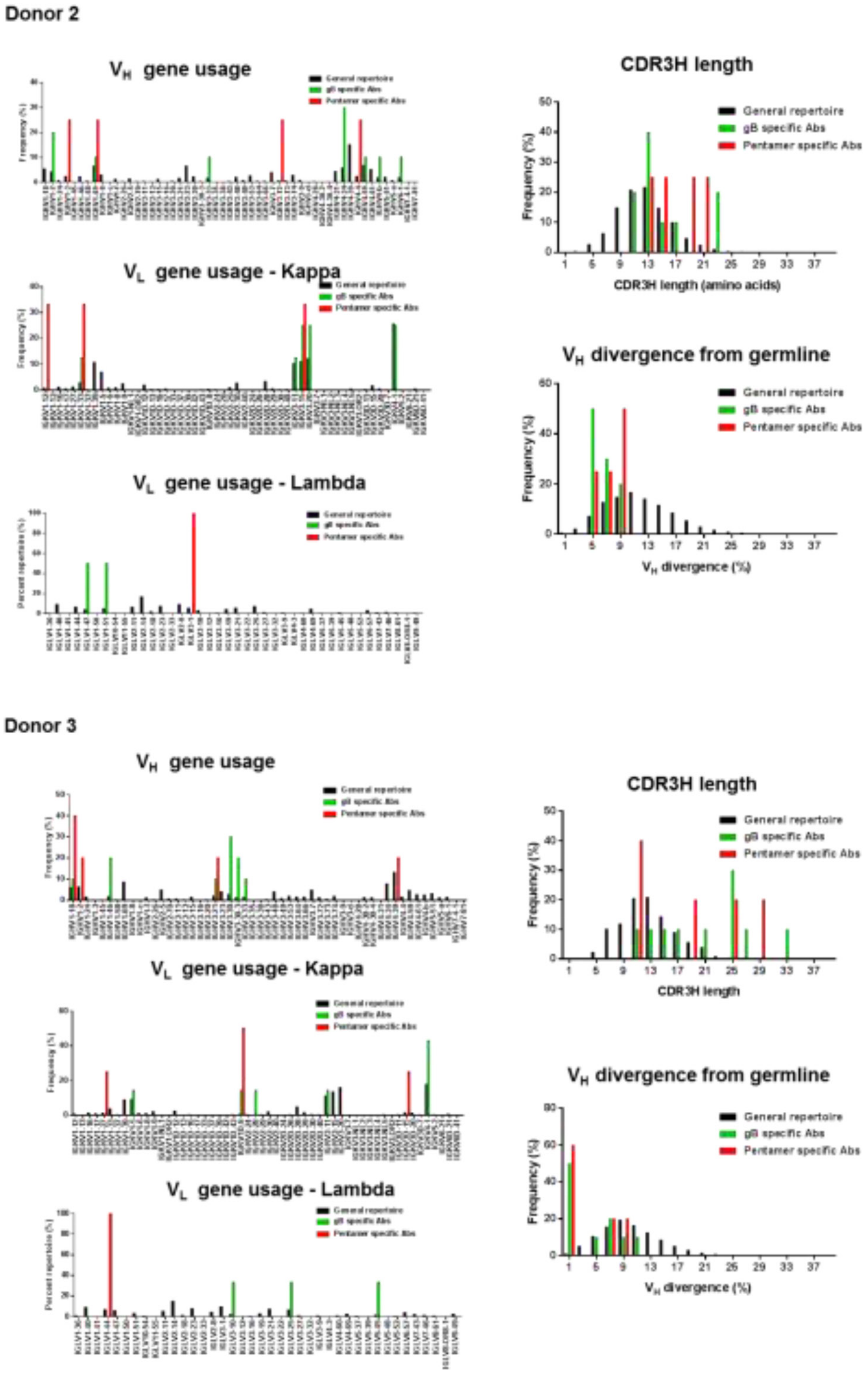

Supplementary Figure 5: Repertoire distributions of donors 2 and 3. For each donor, the processed sequences of PGM sequencing data (black), gB-specific (green) and pentamer-specific sequences (red) of isolated mAbs were respectively used to calculate heavy and light chain repertoire properties such as germline gene usage for heavy chain, kappa chain and lambda chain (left panel), complementarity determining region 3 (CDR3H) length for heavy chain and V_H germline gene divergence (right panel).

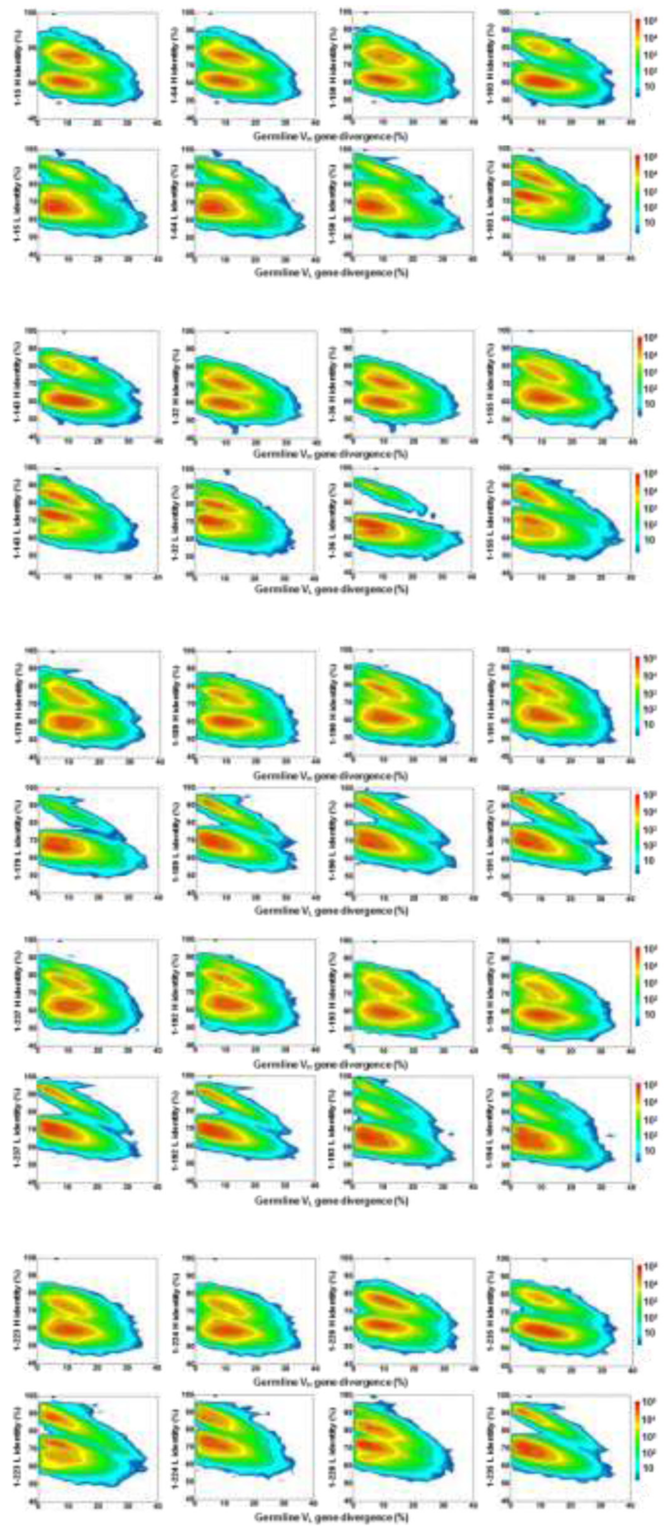

Supplementary Figure 6A: Identity/divergence analysis of the unbiased heavy and light chain repertoires from an HCMV-infected individual, donor 1 (Dn1). The sequences are plotted as a function of sequence identity to the experimentally isolated antibodies except for 1-85, 1-125, and 1-175, which are shown in Figure 4, and sequence divergence from putative germline V genes. Color coding denotes sequence density.

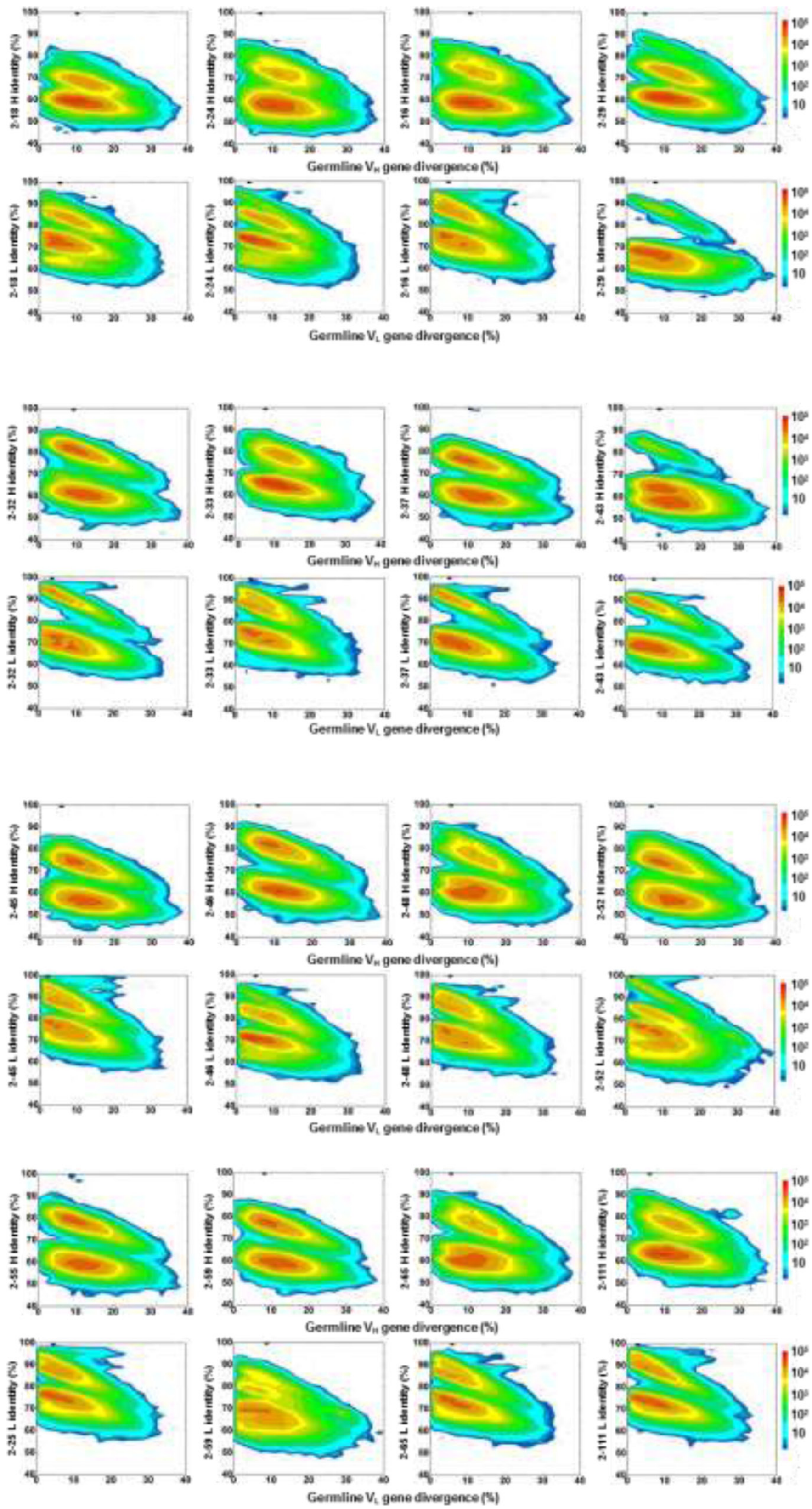

Supplementary Figure 6B: Identity/divergence analysis of the unbiased heavy and light chain repertoires from an HCMV-infected individual, donor 2 (Dn2).

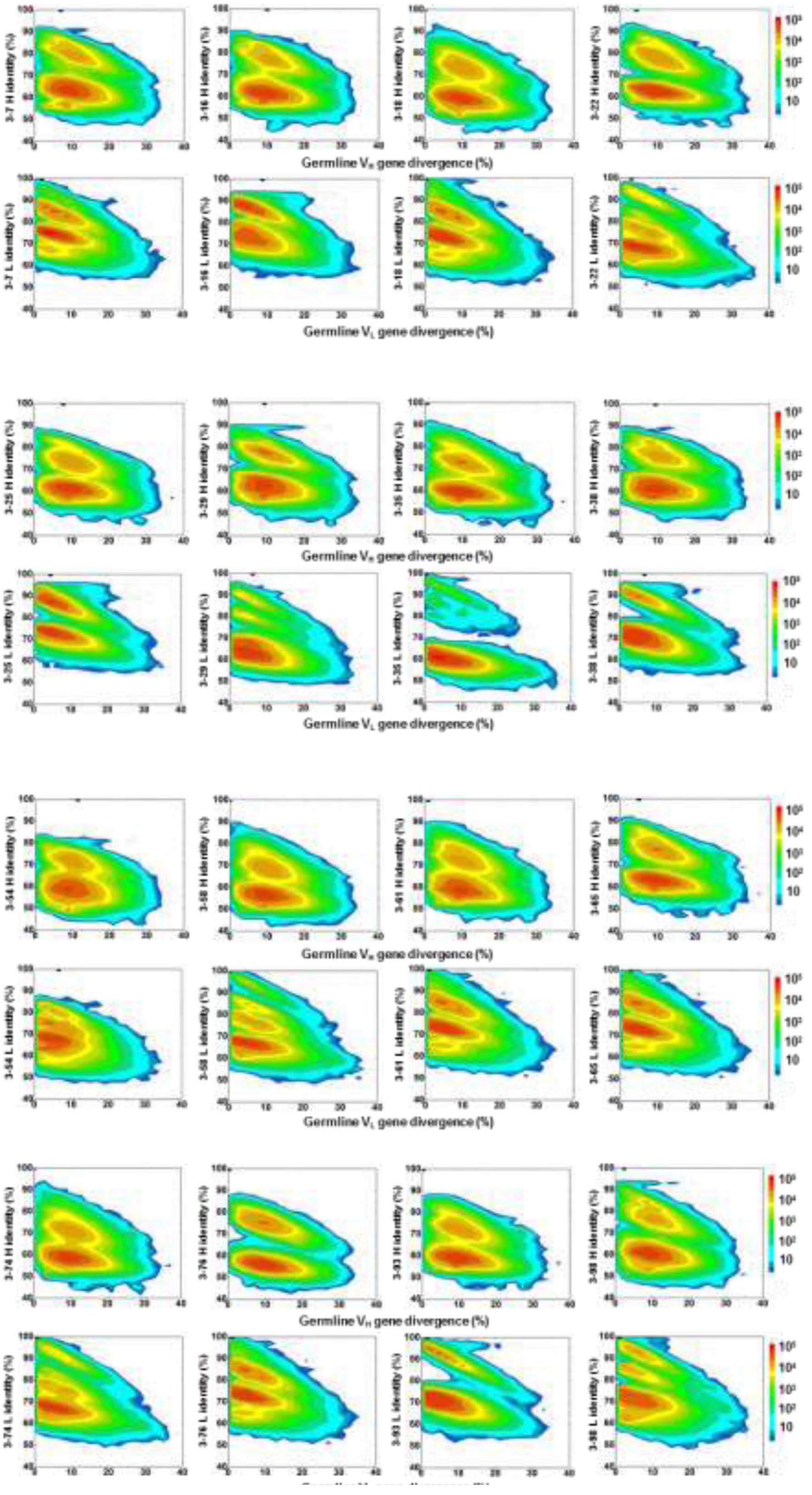

Supplementary Figure 6C: Identity/divergence analysis of the unbiased heavy and light chain repertoires from an HCMV-infected individual, donor 3 (Dn3).

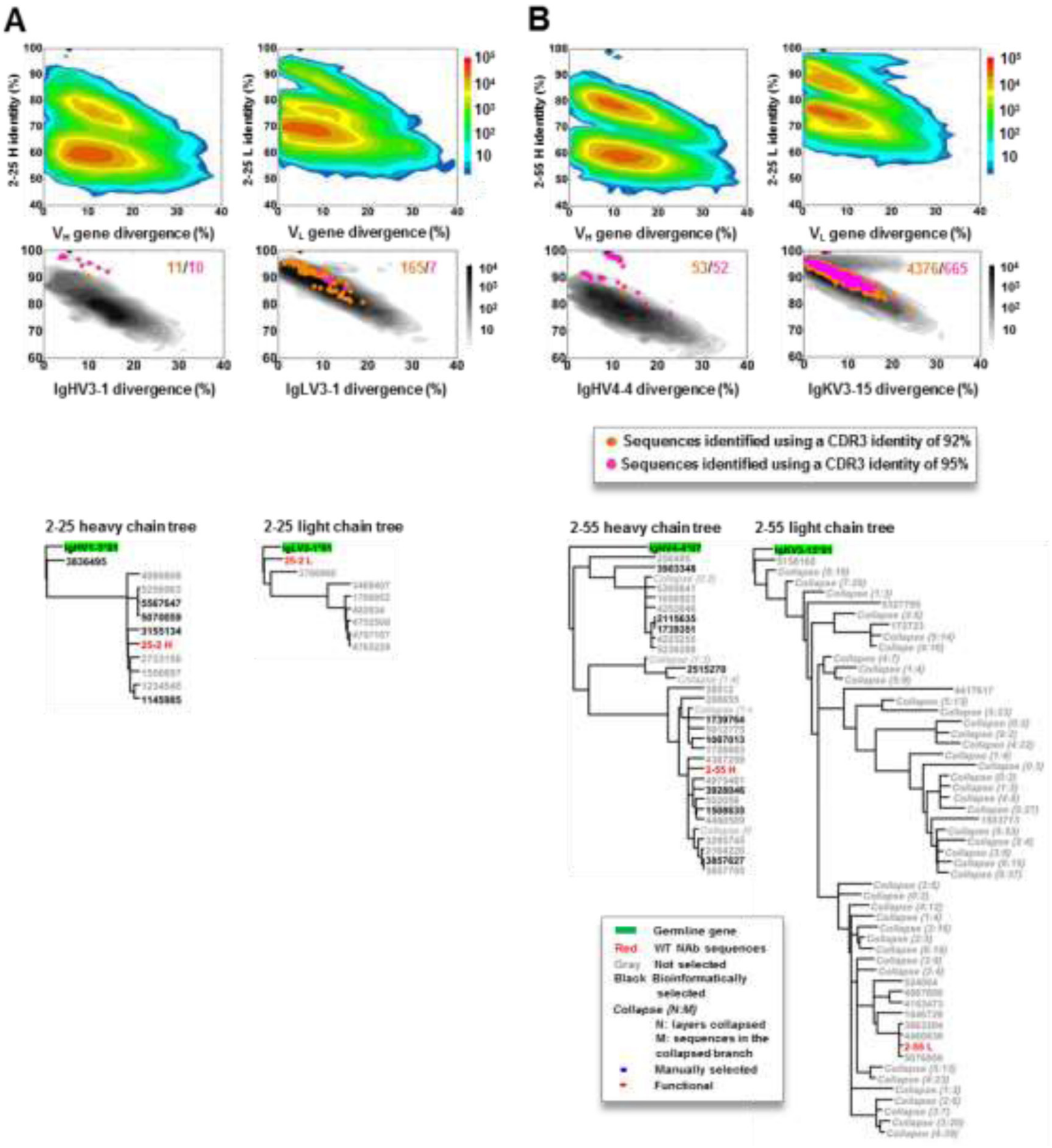

Supplementary Figure 7: Identity/divergence analysis and phylogenetic analysis of the somatic populations of two HCMV-specific antibodies, 2-25 (A) and 2-55 (B), isolated from donor 2 (Dn2). In panel #1, repertoire sequences are plotted as a function of sequence identity to the template antibody chains and sequence divergence from putative germline V genes. Color coding denotes sequence density. In panel #2, sequences with the same germline origin as the template antibody chain are plotted as black contour lines, with those having a CDR3 identity of ≥92% (original dots) and ≥95% (magenta dots) labeled on the 2D plot. In panel #3, heavy and light chain trees derived from the sequences with a CDR3 identity of 95% or greater are constructed for antibodies 2-25 and 2-55. Synthesized sequences are shown in black on the maximum-likelihood (ML) trees.

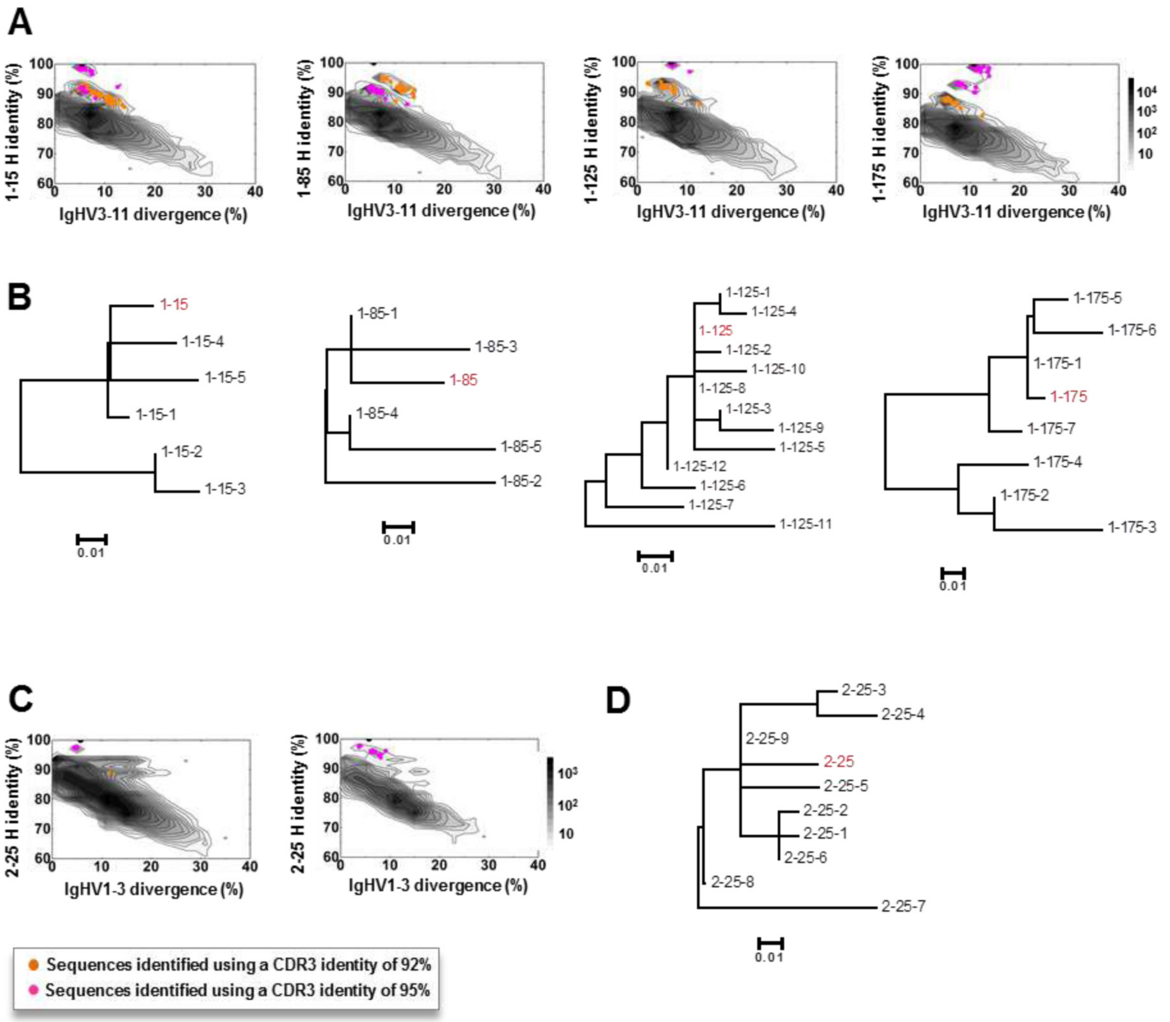

Supplementary Figure 8: Identity/divergence analysis and phylogenetic analysis of the selected genetic variant clones isolated from donor 1 (A, B) and donor 2 (C, D). Repertoire sequences from donor 1 (A) and donor 2 (C) are plotted as a function of sequence identity to the template antibody chains and sequence divergence from putative germline V genes. Color coding denotes sequence density. Sequences with the same germline origin as the template antibody chain are plotted as black contour lines, with those having a CDR3 identity of ≥92% (original dots) and ≥95% (magenta dots) labeled on the 2D plot. Maximum-likelihood (ML) tree were constructed using heavy chain sequences of selected somatic variants from donor 1 (B) and donor 2 (D) with a CDR3 identity of 95% or greater to the template antibody. Synthesized sequences and templated antibody are shown respectively in black and red on the maximum-likelihood (ML) trees.

SUPPLEMENTARY METHODS

Memory B-Cell Culture

A total of 80 mL of whole blood from each donor was collected and processed to peripheral blood mononuclear cells (PBMC) isolation. Briefly, 20 mL of whole blood were added to each 50 mL Accuspin tube (Sigma) pre-filled with 10 mL Hank's solution and centrifuged at 800 x *g* for 15 minutes at room temperature. The upper plasma portion was removed by aspiration and the mononuclear cell band was transferred to a new 50 mL conical tube. The tubes were filled with Hank's solution and centrifuge at 500 x g for 10 minutes at room temperature. The supernatants were discarded and red blood cells in the pellets were lysed by incubation with 5 mL ACK lyse buffer (Gibco Invitrogen) at room temperature for 5 minutes. Afterwards, cell pellets were washed once with 50 ml Hank's solution and re-suspended in 15 mL R10 medium (RPMI 1640 medium with 20% FBS).

IgG^+ memory B-cells were enriched using the customized EasySep™ Human Memory B-Cell Isolation Kit containing anti-human IgM and IgD antibodies (StemCell). Briefly, PBMC was re-suspended in RoboSep buffer to $5x10^7$ cells/mL in a 5 mL polystyrene round bottom tube. EasySep human memory B-cell enrichment cocktail added at 50µl/mL cells. Following 10 minute incubation at room temperature, EasySep D magnetic particles were added at 75 µl/mL. After additional 5 minute incubation at room temperature, RoboSep buffer was added to the cell suspension up to a total volume of 5 mL. Cells were gently mixed in the tube. The tube was placed in the magnet and set aside for 10 min. Supernatant was poured into a new 5 mL polystyrene tube and placed in the magnet for 5 minutes. The procedure was repeated one more time. Supernatant was then poured into a 15 mL conical tube filled with R10 media up to 15 mL. After centrifugation at 200 x g at room temperature for 10 minutes, supernatant was removed and cells were re-suspended in 0.5 mL of R10 medium. Enriched memory B-cells were then seeded at an average of 1.4 B-cells/well in 96-well U-bottom plates in the presence of 40,000 cells/well of gamma-irradiated 293 cells expressing human CD40 ligand and 50 ng/ml of recombinant human IL-21 (Sino Biological). A total of 200 plates were set-up for each donor and cells were cultured for 14 days in the incubator with 5% CO_2 and 90% humidity. Plates were then centrifuged at 2000 rpm for 15 min and supernatants were carefully transferred into new 96-well U-bottom plates without disturbing the cell pellets. Supernatant containing plates were kept at -80°C till screened. The cell pellets were preserved in 50 µL RLT (Qiagen) supplemented with 1% β-mercaptoethanol (Sigma) and stored at -80°C for future RNA isolation.

Screening of Memory B-cell Culture Supernatants

B-cell culture supernatants were first tested for HCMV virion binding and neutralization activities. Samples from the positive hit wells, if there are still supernatants left, were then tested for binding to recombinant HCMV gB and pentameric gH complex proteins, as described previously [1,2]. Briefly, for ELISA assays, antigens were immobilized at 1-2µg/mL in PBS on 96-well Nunc MaxiSorp Immuno plates (ThermoFisher Scientific) at 4°C overnight. Plates were blocked with 3% (vol/vol) nonfat milk in PBS/0.05% Tween-20 and then incubated with undiluted culture supernatants in 40-50 µL volume. Plates were washed afterwards and then HRP-conjugated goat anti-human IgG was added (Southern Biotech, Birmingham, AL) at a dilution of 1:2000. Plates were washed and developed with a HRP substrate, TMB (3,3',5,5'-tetramethylbenzidine) solution (Virolabs) at 100 µL per well for 3 to 5 min. The reaction was stopped by adding 100 µL per well of 1M H_2SO_4 (Sigma). Plates were read at OD 450nm on VICTOR III Multilabel Counter (PerkinElmer). A sample with OD450 reading equal or greater than

0.1 was scored as positive for that well. For neutralization assay, 50µl of supernatants were mixed with 25 µL of AD169rev (~200 pfu) and incubated at room temperature for 1h. The mixture was then transferred into 96-well flat plates which were pre-seeded with ARPE19 cells at 15,000 cells per well one day before. Plates were incubated at 37°C, 5% CO_2 with 90% humidity for 24h and then stained for the expression of immediate early proteins (IE). The total fluorescence intensity was acquired on Li-Cor Odyssey (LI-COR Biosciences). The wells with >80% reduction in IE expression were scored as neutralization positive hits.

To estimate the concentration of IgG in B cell culture supernatants (Figure S1B), supernatants from 8-10 plates were randomly chosen for testing in human IgG ELISA assay. Briefly, Nunc Maxisorp Immuno Plates were coated with 50 µL per well of goat anti-human IgG (Fc) antibody (ThermoFisher Scientific) at 4 µg/mL in PBS at 4°C overnight. Plates were washed in PBS/0.05% Tween 20 and then blocked in 3% non-fat milk/PBS at room temperature for 60 min. Blocking buffer was removed and 50 µL per well of B cell culture supernatants was added. Pates were incubated at room temperature for 90 min and washed in PBS/0.05% Tween 20. Afterwards, 50 µL per well of HRP-conjugated goat anti-Human IgG (Southern Biotech) at 1:2,000 dilution was added. Plates were incubated at room temperature for 60 min and washed. TMB solution was then added (100 µL per well). Reaction was stopped 5 min later with 100 µL per well of 1M H_2SO_4. Plates were read at

450nm on VICTOR III Multilabel Counter.

Cloning and Analysis of the VH and VL Encoding Genes from Memory B-Cells

Cloning of the V_H and V_L encoding genes from single human memory B cells was based on a protocol established in our group for cloning of V_H and V_L encoding genes from single monkey plasma B cells with modifications [3]. Total RNA was isolated from lysed B-cell cultures using the RNeasy Mirco kit (Qiagen). Reverse transcription was carried out using the SuperScript III First-Strand Synthesis SuperMix kit (Invitrogen). Genes encoding IgG heavy and light chain variable regions were amplified by PCR using V_H, V_k, V_λ family-leader region specific primers (Figure S2), using 3.5 µL cDNA template. All PCR reactions were performed in a total volume of 25 µL containing a mixture of 20nM for each primer, 300nM dNTP, 5µL 5X buffer and 1U Primerstar GXL (Clontech) with 5 min incubation at 94°C for the first cycle and followed by 40 cycles of 98°C, 10s; 55°C, 30s; and 68°C, 30s; and a final elongation step at 68°C for 5 min before cooling to 4°C. One microliter of the PCR product was then used as DNA template in a second round of PCR for the generation of DNA fragment for in-frame cloning of the V_H and V_L encoding genes in the IgG expression vectors by infusion cloning. The second round PCR products were run on 2% agarose gels and the 400bp V_H and V_L encoding gene bands were cut and purified for infusion PCR. Infusion PCR was carried out using the In-Fusion® HD Cloning kit (Clontech). Briefly, 50ng of purified PCR products was added to the In-Fusion PCR reaction containing 2 µL In-Fusion HD enzyme premix and 100ng linearized vector with water added up to a total volume of 10 µL. The reaction was carried out by incubation at 50°C for 15 minutes and then stopped on ice. 4 µL of the In-Fusion PCR product was then used for TOP10 competent *E. coli* transformation.

Antibody Sequence Evaluation

Five colonies for each clone from the TOP10 competent *E. coli* transformation were picked for sequencing. Vκ, Vλ and V_H gene sequences were analyzed using GeneBank IgBLAST to identify germline V(D)J gene segments. Individual Vκ, Vλ and V_H genes were mapped to germline genes in the major IGL and IGH locus [4]. Framework and CDR sequences were annotated by following the IMGT (http://www.imgt.org/) nomenclature. Sequence alignment and phylogenetic analysis were performed using CLUSTALX2 (Center for Evolutionary Medicine) [5] and MEGA 5 (Center for Evolutionary Medicine) [6], respectively.

Expression of Human Monoclonal Antibodies

Expression of antibodies in mammalian cells and purification by Protein A/G were described previously [7,8]. Briefly, equal molar amounts of heavy-chain and light-chain plasmids were co-transfected into HEK293F cells for transient expression of antibodies. Supernatants were harvested 7 days later and subjected to testing in ELISA binding and neutralization assays as described above. Those mAbs with confirmed activities were selected for scale-up expression. IgGs were purified with Protein A beads (GE Healthcare) according to the manufacturer's instructions and concentrations were determined using Nanodrop (ThermoFisher Scientific). The purified mAbs were then re-tested in the antigen binding and viral neutralization assays.

Sample Preparation for PGM Sequencing

The 5'-RACE PCR protocol developed for unbiased antibody repertoire capture [9] has been modified to improve the template preparation. Briefly, total RNA was extracted from 20 million PBMCs into 30 µl of water with RNeasy Mini Kit (Qiagen). For unbiased antibody repertoire analysis, 5'-RACE was performed with SMARTer RACE cDNA Amplification Kit (Clontech). The immunoglobulin PCRs were set up with Platinum Taq High-Fidelity DNA Polymerase (Invitrogen Life Technologies, Carlsbad, CA) in a total volume of 50 µl, with 5 µl of cDNA as template, 1 µl of 5'-RACE primer, and 1 µl of 10 µM reverse primer. The 5'-RACE primer contained a PGM P1 adaptor, while the reverse primer contained a PGM A adaptor. 25 cycles of PCRs were performed and the expected PCR products (~600 bp) were gel purified (Qiagen).

Ion Torrent PGM Sequencing of Human Antibody Libraries

We adopted the same PGM sequencing procedure as previously described [9]. Briefly, the antibody heavy (H) and light (κ and λ) chain libraries were quantitated using Qubit® 2.0 Fluorometer with Qubit® dsDNA HS Assay Kit, and then used at a ratio of 1:1:1 for all the PGM sequencing experiments. The dilution factor required for Ion Torrent PGM template preparation was determined such that the final concentration was 50 pM. Template preparation was performed with the

isothermal amplification (IA) Kit obtained from the Early Access Program. Prior to PGM sequencing, quality control of the template was determined by the Qubit® 2.0 Fluorometer with the Ion Sphere™ Quality Control Kit. Sequencing was performed on the Ion Torren PGM sequencer with the PGM™ Hi-Q 400 Kit using an Ion 318 v2 chip for a total of 1200 nucleotide flows. Raw data was processed without the 3'-end trimming in base calling in order to extend the read length [9].

Bioinformatics Analysis of Antibody Sequencing Data

The *Antibodyomics 1.0* pipeline was used to analyze the HCMV antibody repertoires [9-14]. Given an antibody NGS dataset, each sequence was (1) reformatted and labeled with a unique index number; (2) assigned to V, D (for heavy chain only), and J gene families using the current human germline gene database and an in-house implementation of IgBLAST, and sequences with E-value > 10^{-3} for V gene assignment were removed from the data set; (3) subjected to a template-based error-correction procedure, in which insertion and deletion (indel) errors in the V gene segment were detected based on the alignment to their respective germline gene sequences. Note that only indels of less than three nucleotides were corrected; (4) compared to the template antibody sequences at both the nucleotide level and the amino acid level using a global alignment module in CLUSTALW2 [5]; (5) subjected to a multiple sequence alignment (MSA)-based procedure to determine the complementarity determining region 3 (CDR3), which was further compared to the template CDR3 sequences at the nucleotide level, and to determine the sequence boundary of the V(D)J coding region. After full-length variable region sequences were obtained, a bioinformatics filter was applied to detect and remove erroneous sequences that may contain swapped gene segments due to PCR errors. Specifically, a full-length read would be removed if the V-gene alignment was less than 250bp. The processed and annotated antibody sequences were then subjected to a more in-depth repertoire analysis.

REFERENCES FOR SUPPLEMENTARY EXPERIMENTAL PROCEDURES

1. Tang A, Li F, Freed DC, Finnefrock AC, Casimiro DR, Wang D, Fu TM. A novel high-throughput neutralization assay for supporting clinical evaluations of human cytomegalovirus vaccines. Vaccine. 2011; 29:8350–56.
2. Freed DC, Tang Q, Tang A, Li F, He X, Huang Z, Meng W, Xia L, Finnefrock AC, Durr E, Espeseth AS, Casimiro DR, Zhang N, et al. Pentameric complex of viral glycoprotein H is the primary target for potent neutralization by a human cytomegalovirus vaccine. Proc Natl Acad Sci USA. 2013; 110:E4997–5005.
3. Meng W, Li L, Xiong W, Fan X, Deng H, Bett AJ, Chen Z, Tang A, Cox KS, Joyce JG, Freed DC, Thoryk E, Fu TM, et al. Efficient generation of monoclonal antibodies from single rhesus macaque antibody secreting cells. MAbs. 2015; 7:707–18.
4. Malcolm S, Barton P, Murphy C, Ferguson-Smith MA, Bentley DL, Rabbitts TH. Localization of human immunoglobulin κ light chain variable region genes to the short arm of chromosome 2 by in situ hybridization. Proc Natl Acad Sci USA. 1982; 79:4957–61.
5. Larkin MA, Blackshields G, Brown NP, Chenna R, McGettigan PA, McWilliam H, Valentin F, Wallace IM, Wilm A, Lopez R, Thompson JD, Gibson TJ, Higgins DG. Clustal W and Clustal X version 2.0. Bioinformatics. 2007; 23:2947–48.
6. Tamura K, Peterson D, Peterson N, Stecher G, Nei M, Kumar S. MEGA5: molecular evolutionary genetics analysis using maximum likelihood, evolutionary distance, and maximum parsimony methods. Mol Biol Evol. 2011; 28:2731–39.
7. Shi Y, Fan X, Meng W, Deng H, Zhang N, An Z. Engagement of immune effector cells by trastuzumab induces HER2/ERBB2 downregulation in cancer cells through STAT1 activation. Breast Cancer Res. 2014; 16:R33.
8. Fan X, Brezski RJ, Fa M, Deng H, Oberholtzer A, Gonzalez A, Dubinsky WP, Strohl WR, Jordan RE, Zhang N, An Z. A single proteolytic cleavage within the lower hinge of trastuzumab reduces immune effector function and in vivo efficacy. Breast Cancer Res. 2012; 14:R116.
9. He L, Sok D, Azadnia P, Hsueh J, Landais E, Simek M, Koff WC, Poignard P, Burton DR, Zhu J. Toward a more accurate view of human B-cell repertoire by nextgeneration sequencing, unbiased repertoire capture and single-molecule barcoding. Sci Rep. 2014; 4.
10. Wu X, Zhou T, Zhu J, Zhang B, Georgiev I, Wang C, Chen X, Longo NS, Louder M, McKee K, O'Dell S, Perfetto S, Schmidt SD, et al. Focused evolution of HIV-1 neutralizing antibodies revealed by structures and deep sequencing. Science. 2011; 333:1593–602.
11. Zhou T, Zhu J, Wu X, Moquin S, Zhang B, Acharya P, Georgiev IS, Altae-Tran HR, Chuang GY, Joyce MG, Kwon YD, Longo NS, Louder MK, et al. Multidonor analysis reveals structural elements, genetic determinants, and maturation pathway for HIV-1 neutralization by VRC01-class antibodies. Immunity. 2013; 39:245–58.
12. Zhu J, O'Dell S, Ofek G, Pancera M, Wu X, Zhang B, Zhang Z, Mullikin JC, Simek M, Burton DR, Koff WC, Shapiro L, Mascola JR, Kwong PD. Somatic populations of PGT135-137 HIV-1-neutralizing antibodies identified by 454 pyrosequencing and bioinfornnatics. Front Microbiol. 2012; 3:315.
13. Zhu J, Ofek G, Yang Y, Zhang B, Louder MK, Lu G, McKee K, Pancera M, Skinner J, Zhang Z, Parks R, Eudailey J, Lloyd KE, et al. Mining the antibodyome for HIV-1-neutralizing antibodies with next-generation sequencing and phylogenetic pairing of heavy/light chains. Proc Natl Acad Sci USA. 2013; 110:6470–75.

14. Zhu J, Wu X, Zhang B, McKee K, O'Dell S, Soto C, Zhou T, Casazza JP, Mullikin JC, Kwong PD, Mascola JR, Shapiro L, Becker J, et al. De novo identification of VRC01 class HIV-1-neutralizing antibodies by next-generation sequencing of B-cell transcripts. Proc Natl Acad Sci USA. 2013; 110:E4088–97.
